# Digital and Out-of-Facility Care-Seeking Pathways After Acute Respiratory Infection Symptoms in China: Multicity Population-Based Cross-Sectional Study

**DOI:** 10.2196/96371

**Published:** 2026-09-30

**Authors:** Ting Zhang, Jinzhao Cui, Chao Zhang, Xiaochen Zhang, Yongtao Chi, Dazhu Huo, Shengjie Lai, Jianxing Yu, Yu Yang, Zhongjie Li, Weizhong Yang

**Affiliations:** 1 School of Population Medicine and Public Health Chinese Academy of Medical Sciences & Peking Union Medical College Beijing China; 2 Public Health Emergency Management Innovation Center Beijing China; 3 State Key Laboratory of Respiratory Health and Multimorbidity Beijing China; 4 Chinese Field Epidemiology Training Program (CFETP) Beijing China; 5 School of Public Health Shandong Second Medical University Weifang, Shandong China; 6 School of Geography and Environmental Science University of Southampton Southampton, England United Kingdom

**Keywords:** acute respiratory infection, in-person facility attendance, digital health engagement, cross-sectional study, telemedicine

## Abstract

**Background:**

Digital technologies increasingly shape responses to acute respiratory infection (ARI) symptoms through information seeking, consultation, medication purchase, monitoring, and access to diagnostic products or services. However, how digital health engagement overlaps with in-person facility attendance and whether digital and other out-of-facility behaviors provide complementary signals for episodes that are less visible in routine facility-based surveillance remain unclear.

**Objective:**

This study estimates the distribution of ARI episodes by in-person facility attendance and digital health engagement, describes digital and nondigital out-of-facility actions and the reported ordering of these actions, and examines subgroup differences.

**Methods:**

We conducted a population-based cross-sectional survey in 11 Chinese cities using multistage stratified random sampling. Participants reported ARI symptoms within the preceding 14 days and subsequent health-related actions. The primary digital composite included online health information seeking, AI-assisted consultation, telemedicine, online medication purchase, wearable device–based self-monitoring, digitally accessed self-administered rapid antigen testing, and at-home pathogen specimen collection. Participants were cross-classified by in-person facility attendance and digital health engagement. Weighted descriptive proportions were estimated using poststratification weights, with 95% CIs calculated using Wilson score intervals based on the Kish effective sample size. Behavioral sequences were descriptively reconstructed from retrospectively reported timing of actions using 1 representative action per reported day, with adjacent-transition CIs estimated by participant-level bootstrap resampling. Modified Poisson regression with individual-level robust variance estimation was used to estimate adjusted associations with in-person facility attendance, expressed as adjusted prevalence ratios (aPRs).

**Results:**

Among 247,530 participants, 68,650 reported ARI symptoms. Among symptomatic participants, 19.18% (95% CI 18.87%-19.48%) reported in-person facility attendance, and 18.94% (95% CI 18.64%-19.24%) reported at least one digital health behavior. Digital health engagement without in-person facility attendance comprised 11.20% (95% CI 10.95%-11.44%) of ARI episodes, while 7.74% (95% CI 7.54%-7.95%) involved both dimensions. Digital health engagement was positively associated with in-person facility attendance during the same episode (aPR 2.12, 95% CI 2.05-2.20). Compared with participants reporting 2 or fewer symptoms, the aPRs were 3.31 (95% CI 3.16-3.46) for 3 symptoms and 4.69 (95% CI 4.51-4.88) for 4 or more symptoms. Among participants with nonempty sequences, the most common sequence beginning with an out-of-facility action was offline medication purchase followed by no subsequent reported action (6058/26,494, 22.87%). Digital health engagement was lowest among adults aged 60 years or older.

**Conclusions:**

Most reported ARI episodes did not involve in-person facility attendance and may be underrepresented in routine facility-based surveillance. Digital health behaviors identified potential complementary signals for some episodes, while common pathways involving offline medication purchase suggest that pharmacy-derived data may provide additional information. These data sources require direct validation, and lower digital health engagement among older adults indicates that digital signals may not represent all population groups equally.

## Introduction

Acute respiratory infections (ARIs) are a major cause of morbidity worldwide and continue to place substantial pressure on health systems, particularly during seasonal epidemics and periods of concurrent respiratory virus circulation [1,2]. In populous settings such as China, recurrent surges in respiratory infection symptoms can rapidly increase demand for consultation, testing, and treatment, while also complicating the timely interpretation of disease activity at the population level [2,3]. Understanding how symptomatic individuals respond following illness onset is therefore important for estimating health care demand, calibrating facility-based surveillance data, interpreting digital health signals, and identifying groups that may remain underrepresented in routine data streams [3-6].

Routine respiratory infectious disease surveillance in China relies substantially on clinical, syndromic, and laboratory information generated through health care facilities, including sentinel hospitals, outpatient and emergency departments, fever clinics, and clinical laboratories [2,7]. Consequently, community ARI episodes that do not involve in-person health care facility attendance may be underrepresented in routinely collected facility-based data [4-6]. These episodes constitute a less visible component of the community illness burden, although some may generate behavioral signals through digital platforms or other out-of-facility channels [7-10].

The expansion of digital technologies is reshaping how individuals respond to respiratory symptoms. Drawing on the World Health Organization’s conceptualization of digital health [11,12], we considered digital health behaviors to be health-related actions undertaken, delivered, supported, or enhanced through digital technologies. These behaviors may facilitate access to health information, automated or clinician-provided advice, medicines, diagnostic products or services, and self-monitoring information without necessarily requiring an in-person visit to a health care facility [11,12].

Two testing-related behaviors require specific clarification in the Chinese context. Self-administered rapid antigen testing involves individuals obtaining a test kit, collecting their own specimen, performing the test, and interpreting the result themselves. Test kits may be purchased through smartphone-based e-commerce or on-demand delivery platforms, but may also be purchased from offline pharmacies. At-home pathogen specimen collection is a separate service in which individuals book testing through a mobile platform, trained personnel collect respiratory specimens at home, and the specimens are subsequently tested in a laboratory.

Despite growing interest in digital health, the relationship between digital health engagement and in-person facility attendance remains insufficiently characterized [5,6,13]. These dimensions capture different properties of an illness episode: in-person facility attendance reflects whether an individual sought face-to-face care at a health care facility, whereas digital health engagement reflects whether the episode involved a technology-mediated health-related action. They may occur independently or together and should therefore be cross-classified rather than treated as mutually exclusive categories.

This cross-classification also has potential relevance for surveillance interpretation. Episodes involving in-person facility attendance may contribute to facility-derived clinical data, whereas episodes with digital health engagement but without in-person facility attendance may remain outside facility-based data while potentially generating signals through digital health behaviors [3,7-10,13]. Episodes involving neither measured dimension may be less visible through either source, although they may still involve offline medication purchase, offline self-testing, or other unmeasured forms of self-management [14].

In this study, we used data from a large population-based survey conducted across 11 cities in China to characterize responses to ARI symptoms within the preceding 14 days. Our primary analytical framework cross-classified each ARI episode according to 2 dimensions: whether the individual attended a health care facility in person and whether the individual engaged in any digital health behavior. We aimed to (1) estimate the distribution of the 4 mutually exclusive groups defined by these dimensions; (2) describe specific digital and nondigital actions, including online health information seeking, AI-assisted consultation, telemedicine, online and offline medication purchase, wearable device–based self-monitoring, self-administered rapid antigen testing, and at-home pathogen specimen collection; (3) characterize the reported ordering of these actions; and (4) examine subgroup differences in digital health engagement and in-person facility attendance. By jointly examining digital health engagement and in-person facility attendance, this study provides a population-level description of how community ARI episodes are distributed across behavioral pathways with different potential visibility in facility-based and digital data sources.

## Methods

### Study Design and Population

We conducted a population-based cross-sectional survey from January 13 to 27, 2025, using a multistage stratified random sampling design across the study cities. The 11 study cities were randomly selected from the official national registry of cities in China. The cities covered eastern, central, western, northeastern, and southwestern China and represented different population sizes and levels of economic development.

Within each city, multistage stratified random sampling was conducted. Based on city statistical yearbooks, districts/counties were classified into high, medium, and low levels of health care resources using an index calculated as the number of hospital beds per 1000 population multiplied by the number of health care institutions per 1000 population. Districts/counties were selected using city-specific sampling procedures, and the number participating varied across study sites. Within the sampled districts/counties, subdistricts, townships, or towns were sampled from the applicable administrative categories using official administrative lists. Communities/villages were then selected by simple random sampling from complete rosters provided and verified by local administrative authorities and Centers for Disease Control and Prevention. Within each community/village, all households were listed and numbered from 1 to N, and households were selected using systematic sampling. All eligible members of selected households were invited to participate. If an age group was underrepresented because of refusal or nonresponse, additional households were sampled from the same area using the same procedure.

Trained field investigators contacted selected households and provided standardized instructions. The questionnaire was completed electronically, with assistance provided when a participant was unable to complete it independently. During fieldwork, sites monitored recruitment against prespecified age-group and urban-rural sample targets. When these targets were not met because of refusal, nonresponse, or insufficient accrual within the initially selected units, sites could add counties, districts, communities, or households. At each sampling stage, additional units were randomly selected from the remaining eligible units using the same procedure as for the initial selection, and the additions were documented separately. The target sample size was 200,486 and was increased to 220,535 to allow for anticipated nonresponse.

Eligible participants were community residents of all ages. Participants who were able to complete the questionnaire independently were asked to respond for themselves. There was no fixed age cutoff for proxy completion. For participants unable to complete the questionnaire independently, including young children and some older or otherwise dependent household members, a parent, legal guardian, adult child, or other household member could respond on the participant’s behalf. Participants younger than 18 years required permission from a parent or legal guardian.

The 18-59-year age group was adopted to align with China’s National Population Census calibration categories used for poststratification weighting. Sex was recorded as a binary variable (male or female), consistent with national census categories; gender identity was not assessed. Participants were asked whether they had experienced ARI symptoms during the preceding 14 days. ARI was identified using standardized symptom-based criteria, and influenza-like illness (ILI) was defined as fever plus cough or sore throat. For participants aged 2 years or older, ARI was defined using age-appropriate symptom combinations; for children younger than 2 years and nonverbal participants, ARI was defined based on guardian-reported respiratory symptoms.

### Definitions of Care-Seeking and Digital Health Behaviors

In-person facility attendance was defined as any face-to-face visit to a formal health care institution during an ARI episode, including hospitals, community health centers, outpatient clinics, emergency departments, and other licensed medical facilities. This measure constituted the first dimension of the primary participant-level framework and indicated whether the ARI episode involved facility-based care. Offline medication purchase was defined separately as the in-person purchase of medicines from a community or retail pharmacy and was treated as a nondigital, out-of-facility action rather than as an additional dimension of the primary framework.

Drawing on the World Health Organization’s conceptualization of digital health, we operationally defined digital health behaviors as health-related actions undertaken, delivered, supported, or enhanced through digital technologies during an ARI episode. Digital mediation included internet-based platforms, smartphone apps, artificial intelligence systems, telecommunication services, e-commerce or on-demand delivery platforms, and connected monitoring devices. The 7 digital health behaviors included in the primary definition were online health information seeking, AI-assisted consultation, telemedicine, online medication purchase, wearable device–based self-monitoring (wearable monitoring), self-testing using a kit obtained through an e-commerce or on-demand delivery platform, and at-home pathogen specimen collection.

Self-administered rapid antigen testing (self-testing) referred to participants obtaining a rapid antigen test kit, collecting their own respiratory specimen, performing the test, and interpreting the result themselves. The questionnaire distinguished among kits obtained from offline pharmacies, e-commerce platforms, and on-demand delivery platforms. In the primary channel–based definition, self-testing contributed to digital health engagement only when the kit was obtained through an e-commerce or on-demand delivery platform. Self-testing using a kit obtained exclusively from an offline pharmacy was classified as a nondigital, out-of-facility action unless the participant reported another digital health behavior.

At-home pathogen specimen collection (at-home pathogen) was distinguished from self-testing. It referred specifically to a service booked through a mobile platform, after which trained personnel visited the participant’s home to collect a respiratory specimen for laboratory-based pathogen testing. As the service was booked and coordinated digitally, all reported use of at-home pathogen specimen collection was classified as a digital health behavior in the primary analysis.

In a sensitivity analysis, we applied a broader definition in which all self-administered rapid antigen testing was considered digital, regardless of the acquisition channel. We also evaluated 2 extreme scenarios for ambiguous acquisition channels to assess whether channel ambiguity materially affected estimates of digital health engagement and the 4 participant-level groups.

Participants could report multiple actions during the same ARI episode; therefore, action-level behaviors were not mutually exclusive. The primary participant-level framework did not assign every reported action to a single behavioral category. Instead, it cross-classified participants according to 2 binary dimensions: (1) whether they reported in-person facility attendance and (2) whether they engaged in at least 1 of the 7 digital health behaviors. As these dimensions represented different aspects of an illness episode—care setting and digital mediation—they could overlap.

This cross-classification generated 4 mutually exclusive groups: (1) in-person facility attendance with digital health engagement, (2) in-person facility attendance without digital health engagement, (3) digital health engagement without in-person facility attendance, and (4) neither in-person facility attendance nor digital health engagement. The fourth group should not be interpreted as having taken no action. Participants in this group could still have purchased medications offline, used self-testing kits obtained from offline pharmacies, practiced home-based self-management or watchful waiting, or taken no action. These nondigital, out-of-facility behaviors were analyzed separately.

### Data Collection and Quality Control

Data were collected through face-to-face household surveys using an electronic questionnaire administered on mobile devices. Trained investigators visited the preselected households, screened potential participants for eligibility, explained the study objectives and procedures, and obtained written or electronic informed consent before collecting data. Children, older adults, and other participants who were unable to respond independently could receive assistance from a parent, legal guardian, adult child, or other household member.

To ensure consistency across study sites, all investigators received standardized training before fieldwork. Quality-control procedures included real-time logic and range checks, standardized field supervision, and centralized review of data completeness and internal consistency. Although proxy-assisted responses were permitted, a proxy-response indicator was not retained in the analytic database; therefore, these responses could not be separately identified in the analysis. Consequently, response mode could not be accounted for or examined in sensitivity analyses. This limitation is particularly relevant to young children and some older or dependent adults, whose digital health activities may have been performed, facilitated, observed, or reported by another household member. The complete questionnaire, including the original Chinese version and its English translation, is provided in Multimedia Appendix 1.

### Reconstruction of Reported Behavioral Sequences

For participants reporting recent ARI symptoms, reported actions were ordered by the number of days since symptom onset. Each action-level behavior was treated as a distinct state. When multiple actions were reported on the same calendar day, 1 representative state was assigned using a prespecified hierarchy based on the level of clinical involvement. This rule was used only to create an analyzable daily sequence and did not imply that 1 action preceded or caused another when both occurred on the same day. As the questionnaire recorded timing by day only, within-day action order was unavailable. Collapsing multiple actions reported on the same day into a single representative state removed co-occurrence information and, by representing each day with 1 state, imposed an artificial ordering that could affect the frequencies of states, complete sequences, and adjacent transitions. The resulting sequences therefore represent a rule-based daily simplification rather than the complete order in which all actions occurred.

Only transitions between adjacent dated states were counted. A terminal state, “no subsequent reported action,” was appended to every nonempty sequence. This terminal state indicated only that no later dated action was recorded in the questionnaire; it did not indicate symptom resolution, deliberate watchful waiting, or successful self-management. Episodes with missing or nonorderable timing information were excluded from sequence-based analyses but retained in descriptive analyses.

We described the number of dated states and transitions contributed by each participant. CIs for transition probabilities were estimated using 2000 participant-level bootstrap replicates, in which all transitions from a selected participant were retained together. The analysis was described as an empirical adjacent-transition analysis rather than a first-order Markov model because it summarizes reported ordering without assuming a generative Markov process. A sensitivity analysis excluding participants who reported all actions on the same day yielded substantively similar results. However, this sensitivity analysis could not recover the true within-day ordering of actions. Additional details on behavioral sequence reconstruction, same-day action prioritization, eligibility, and transition estimation are provided in Methods S1 in Multimedia Appendix 2.

### Statistical Analysis

Descriptive analyses incorporated poststratification weights calibrated by iterative proportional fitting to age group, sex, and urban-rural residence distributions from the latest national population census. Weighted estimates describe the sampled population after demographic calibration and should not be interpreted as nationally representative estimates unless all stage-specific selection probabilities are incorporated into the analysis weights.

Weighted 95% CIs for descriptive proportions were calculated using Wilson score intervals based on the Kish effective sample size derived from the poststratification weights within each analytic subgroup.

As in-person facility attendance was not a rare outcome, modified Poisson regression with a log link and individual-level robust (sandwich) variance estimation was used to estimate adjusted prevalence ratios (aPRs) and 95% CIs.

The overall strict digital health engagement model adjusted for age group, sex, urban-rural residence, regional economic level, smartphone ownership, and symptom-count category. Marginal standardization averaged model predictions over the observed weighted covariate distribution to estimate the adjusted prevalence of in-person facility attendance and its difference.

Model specification was based on the exposure or research question rather than applying a single common fully adjusted model to all variables. The primary symptom-burden model included symptom-count category as the exposure and adjusted for age group, sex, urban-rural residence, regional economic level, and smartphone ownership.

Associations with individual symptoms were estimated in a separate model that included the individual symptom indicators and the same demographic covariates but excluded symptom-count category, thereby avoiding simultaneous adjustment for a composite measure and its component symptoms.

Each digital or out-of-facility behavior was examined in a separate episode-level model adjusted for age group, sex, urban-rural residence, regional economic level, smartphone ownership, and symptom-count category. Behavioral exposures were not mutually adjusted for one another because they could form part of the same illness-response pathway.

As behaviors and in-person facility attendance were reported for the same illness episode, these models estimate cross-sectional, episode-level associations. They should not be interpreted as causal effects, predictors of subsequent attendance, or evidence that a behavior delayed or substituted for facility-based care.

Sensitivity analyses counted all self-testing as digital, assigned ambiguous self-testing channels under 2 extreme scenarios, used weighted logistic regression, and split the 18-59-year age group into 18-39 and 40-59 years. All regression sensitivity analyses used individual-level robust variance estimation. Statistical analyses were conducted using R (R Foundation for Statistical Computing). Interpretation focused on association estimates, 95% CIs, and epidemiological relevance rather than *P* values.

### Ethical Considerations

The study protocol was reviewed and approved by the Research Ethics Review Committee for Biomedical Research Involving Humans at the Chinese Academy of Medical Sciences and Peking Union Medical College (approval number CAMS&PUMC-IEC-2025-010). All adult participants provided informed consent before participation. Participants younger than 18 years required permission from a parent or legal guardian. Age-appropriate assent was obtained from minors when applicable under the approved study procedures. There was no fixed age cutoff for proxy completion; proxy response was based on the participant’s ability to complete the questionnaire independently.

## Results

### Study Population and Prevalence of Recent ARI

Among 268,776 residents who consented to and initiated the survey (Figure 1), 247,530 were included in the final analytic dataset; 21,246 were excluded because of incomplete data, failure to meet eligibility criteria, or failure to pass prespecified quality-control checks, corresponding to an analysis exclusion rate of 7.90%. Among included participants, 68,650 reported ARI symptoms during the preceding 14 days, corresponding to a crude prevalence of 27.73% (95% CI 27.56%-27.91%) and a weighted prevalence of 27.40% (95% CI 27.22%-27.59%; Table 1). City-specific survey initiation, analytic inclusion, and exclusion counts are provided in Table S1 in Multimedia Appendix 2.

**Figure 1:**
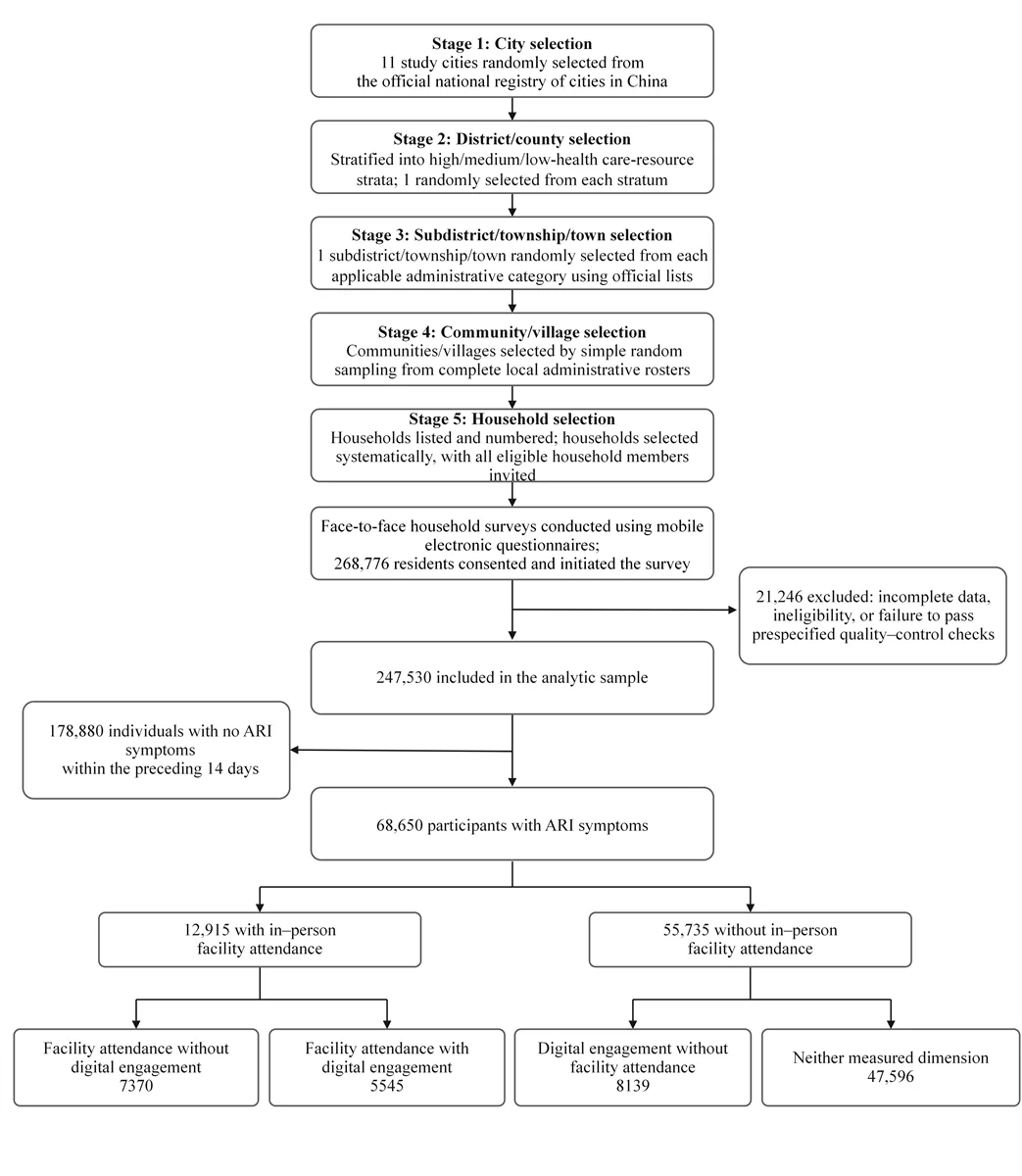
Study profile. The figure shows participant recruitment and analytic sample selection for a cross-sectional survey conducted in 11 Chinese cities from January 13 to 27, 2025. Of 268,776 residents who consented to and initiated the survey, 21,246 were excluded because of incomplete data, failure to meet eligibility criteria, or failure to pass prespecified quality-control checks, leaving 247,530 participants in the analytic sample. Of these, 68,650 reported acute respiratory infection (ARI) symptoms within the preceding 14 days. Among participants with recent ARI symptoms, in-person facility attendance and digital health engagement under the primary channel–based definition were used to classify participants into 4 mutually exclusive groups. Telemedicine was classified as a digital health behavior but not as in-person facility attendance. All counts are unweighted.

**Table 1:** Crude and weighted prevalence of ARI^a^ by demographic and geographic characteristics (N=247,530).

Variable		Participants with ARI symptoms, n	Total surveyed, N	Crude prevalence^b^ (95% CI), %	Weighted prevalence^c^ (95% CI), %
Overall		68,650	247,530	27.73 (27.56-27.91)	27.40 (27.22-27.59)
**Sex**					
	Male	29,905	114,372	26.15 (25.89-26.40)	26.00 (25.74-26.27)
	Female	38,745	133,158	29.10 (28.85-29.34)	28.78 (28.53-29.03)
**Age group (years)**					
	<5	3510	9365	37.48 (36.50-38.46)	37.51 (36.53-38.50)
	5-17	12,043	42,448	28.37 (27.94-28.80)	28.43 (28.00-28.87)
	18-59	45,254	163,785	27.63 (27.41-27.85)	27.46 (27.25-27.68)
	≥60	7843	31,932	24.56 (24.09-25.03)	24.60 (24.13-25.08)
**Residence**					
	Rural	27,844	100,282	27.77 (27.49-28.04)	27.56 (27.28-27.85)
	Urban	40,806	147,248	27.71 (27.48-27.94)	27.28 (27.05-27.51)
**City-level gross domestic product**					
	High	19,341	70,287	27.52 (27.19-27.85)	27.35 (27.01-27.70)
	Middle	37,461	135,768	27.59 (27.35-27.83)	27.18 (26.93-27.42)
	Low	11,848	41,475	28.57 (28.13-29.00)	28.19 (27.74-28.64)
**Smartphone ownership**					
	Yes	53,261	196,430	27.11 (26.92-27.31)	26.75 (26.54-26.95)
	No	15,389	51,100	30.12 (29.72-30.51)	29.81 (29.39-30.22)
**City**					
	Chongqing	5637	15,039	37.48 (36.71-38.26)	38.23 (37.42-39.04)
	Xiangtan	2733	8571	31.89 (30.90-32.87)	32.04 (31.01-33.08)
	Baiyin	4650	13,344	34.85 (34.04-35.66)	34.40 (33.57-35.24)
	Weifang	6418	17,464	36.75 (36.03-37.46)	36.24 (35.51-36.98)
	Yichang	4739	15,195	31.19 (30.45-31.92)	30.69 (29.94-31.45)
	Dalian	13,145	46,430	28.31 (27.90-28.72)	27.69 (27.27-28.11)
	Shanghai	4155	15,737	26.40 (25.71-27.09)	25.29 (24.59-26.00)
	Chengdu	6562	23,004	28.53 (27.94-29.11)	27.96 (27.37-28.57)
	Suining	4465	19,560	22.83 (22.24-23.42)	22.26 (21.66-22.87)
	Shenyang	13,159	56,679	23.22 (22.87-23.56)	22.90 (22.54-23.26)
	Hangzhou	2987	16,507	18.10 (17.51-18.68)	17.39 (16.80-17.99)

^a^ARI: acute respiratory infection.

^b^n/N values are unweighted counts. Crude prevalence is calculated directly as n/N.

^c^Weighted prevalence and its 95% CI are survey-weighted estimates based on poststratification weights; therefore, no directly corresponding n/N is applicable.

ARI prevalence varied across age groups. The weighted prevalence was highest among children younger than 5 years (37.51%, 95% CI 36.53%-38.50%), followed by participants aged 5-17 years (28.43%, 95% CI 28.00%-28.87%) and those aged 18-59 years (27.46%, 95% CI 27.25%-27.68%), and was lowest among adults aged 60 years or older (24.60%, 95% CI 24.13%-25.08%). ARI prevalence was higher among female participants than among male participants (28.78%, 95% CI 28.53%-29.03% vs 26.00%, 95% CI 25.74%-26.27%). Detailed demographic- and city-specific ARI prevalence estimates are provided in Table S2 in Multimedia Appendix 2.

### Cross-Classification of In-Person Facility Attendance and Digital Health Engagement

Under the primary channel–based definition, 18.94% (95% CI 18.64%-19.24%) of participants with recent ARI symptoms reported at least one digital health behavior. Of the 4 mutually exclusive groups, 11.43% (95% CI 11.19%-11.68%) reported in-person facility attendance without digital health engagement, 7.74% (95% CI 7.54%-7.95%) reported both in-person facility attendance and digital health engagement, 11.20% (95% CI 10.95%-11.44%) reported digital health engagement without in-person facility attendance, and 69.63% (95% CI 69.27%-69.98%) reported neither measured dimension (Figure 2).

**Figure 2:**
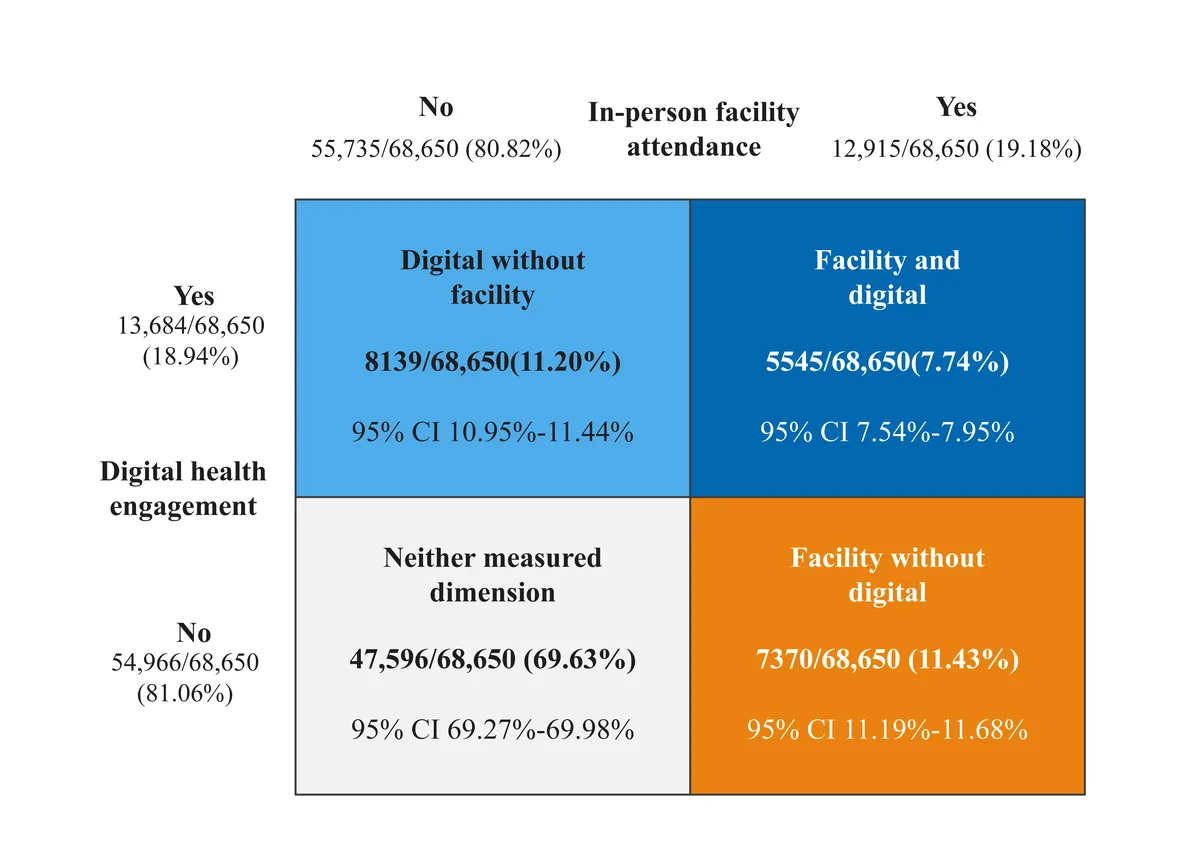
Cross-classification of in-person facility attendance and digital health engagement among participants reporting recent acute respiratory infection symptoms. The 2 dimensions can overlap. Digital health engagement uses the strict primary definition: online health information seeking, AI-assisted consultation, telemedicine, online medication purchase, wearable monitoring, self-testing with a kit obtained through an e-commerce or on-demand delivery platform, or at-home pathogen specimen collection. "Neither measured dimension" does not indicate that no action was taken; this group may include offline medication purchase and self-testing with a kit obtained exclusively from an offline pharmacy. The figure describes measured behavioral dimensions and does not represent actual surveillance coverage or data availability to surveillance authorities. Values are survey-weighted proportions based on poststratification calibration weights; 95% CIs are Wilson score intervals based on the Kish effective sample size.

The final group should not be interpreted as having taken no action. Participants in this group could have purchased medications offline, obtained a rapid antigen test exclusively from an offline pharmacy, or undertaken other actions not included in the digital composite.

Among participants without in-person facility attendance, 13.85% (95% CI 13.56%-14.15%) reported strict digital health engagement. Conversely, 59.11% (95% CI 58.27%-59.95%) of participants with strict digital health engagement did not report in-person facility attendance. These estimates describe potential behavioral visibility rather than actual inclusion in surveillance data.

### Prevalence of Individual In-Person, Out-of-Facility, and Digital Health Behaviors

The weighted prevalence of in-person facility attendance was 19.18% (95% CI 18.87%-19.48%), and the weighted prevalence of offline medication purchase was 19.66% (95% CI 19.36%-19.97%). Among the core digital health behaviors, online health information seeking was most frequent (12.42%), followed by online medication purchase (5.97%), AI-assisted consultation (4.96%), and telemedicine (4.13%). Detailed survey-weighted comparisons across demographic subgroups are shown in Table 2 and Table S3 in Multimedia Appendix 2.

**Table 2:** Distribution of mutually exclusive participant-level groups under the primary channel–based digital health definition, by subgroup (N=68,650; survey-weighted proportions)^a^.

Characteristics			In-person facility attendance without digital health engagement, %		In-person facility attendance with digital health engagement, %		Digital health engagement without in-person facility attendance, %		Neither measured dimension, %
Overall (N=68,650)			11.43 (11.19-11.68)		7.74 (7.54-7.95)		11.20 (10.95-11.44)		69.63 (69.27-69.98)
**Sex**									
	Male	11.70 (11.33-12.08)		6.86 (6.57-7.16)		10.27 (9.92-10.63)		71.17 (70.64-71.70)	
	Female	11.20 (10.88-11.53)		8.53 (8.25-8.82)		12.02 (11.69-12.35)		68.25 (67.77-68.73)	
**Age group**									
	<5 years	17.31 (16.09-18.60)		8.35 (7.47-9.31)		7.07 (6.26-7.97)		67.28 (65.70-68.81)	
	5-17 years	13.20 (12.61-13.82)		6.47 (6.04-6.92)		7.63 (7.17-8.12)		72.70 (71.89-73.49)	
	18-59 years	8.65 (8.39-8.91)		8.96 (8.70-9.23)		14.26 (13.94-14.59)		68.13 (67.69-68.56)	
	≥60 years	17.01 (16.19-17.86)		4.79 (4.34-5.29)		5.65 (5.16-6.19)		72.55 (71.54-73.53)	
**Residence**									
	Urban	9.19 (8.90-9.48)		8.01 (7.74-8.28)		12.42 (12.09-12.75)		70.39 (69.93-70.84)	
	Rural	14.28 (13.85-14.71)		7.41 (7.10-7.73)		9.65 (9.30-10.01)		68.67 (68.10-69.23)	
**City-level** **gross domestic product**									
	High gross domestic product	14.36 (13.85-14.88)		7.76 (7.38-8.16)		10.39 (9.95-10.85)		67.49 (66.80-68.17)	
	Middle gross domestic product	7.65 (7.38-7.93)		7.58 (7.31-7.86)		12.82 (12.47-13.17)		71.95 (71.48-72.42)	
	Low gross domestic product	18.05 (17.34-18.78)		8.20 (7.70-8.73)		7.65 (7.17-8.16)		66.10 (65.21-66.98)	
**Smartphone ownership**									
	Owner	9.94 (9.68-10.20)		8.53 (8.29-8.78)		12.76 (12.47-13.05)		68.77 (68.36-69.18)	
	Nonowner	16.33 (15.73-16.95)		5.17 (4.81-5.54)		6.08 (5.70-6.49)		72.42 (71.68-73.15)	
**Influenza-like illness** **status**									
	Influenza-like illness	25.68 (24.79-26.59)		18.45 (17.66-19.26)		17.62 (16.85-18.42)		38.24 (37.25-39.25)	
	Noninfluenza-like illness	9.10 (8.86-9.34)		5.99 (5.80-6.19)		10.14 (9.89-10.40)		74.77 (74.40-75.13)	

^a^The 4 groups are mutually exclusive at the participant level and are defined by 2 binary dimensions: in-person facility attendance and digital health engagement under the primary channel–based definition. Offline medication purchase, exclusively offline self-testing, and other nondigital out-of-facility actions were analyzed separately and may occur in either of the 2 groups without digital health engagement. The group with neither measured dimension should not be interpreted as having taken no action. Values are survey-weighted percentages with 95% CIs calculated using Wilson score intervals based on the Kish effective sample size derived from poststratification weights within each subgroup.

Among 2203 participants who reported self-administered rapid antigen testing, 1121 (50.89%) obtained test kits exclusively from offline pharmacies, 783 (35.54%) used only e-commerce or on-demand delivery platforms, 260 (11.80%) reported both offline and digital channels, and 39 (1.77%) reported another or missing channel. Overall, 1043 participants (47.34%) reported using at least one digital channel to obtain a self-test kit.

At-home pathogen specimen collection was reported by 915 participants and had a weighted prevalence of 1.29% (95% CI 1.21%-1.38%). When all self-administered rapid antigen testing was classified as digital regardless of acquisition channel, the weighted prevalence of any digital health behavior increased from 18.94% to 19.37% (95% CI 19.07%-19.68%). Under this broader definition, 11.37% (95% CI 11.13%-11.62%) reported digital health engagement without in-person facility attendance, 8.00% (95% CI 7.79%-8.21%) reported both, 11.18% (95% CI 10.94%-11.42%) reported in-person facility attendance without digital health engagement, and 69.45% (95% CI 69.09%-69.80%) reported neither. The substantive interpretation was unchanged.

### Variation in Facility Attendance and Digital Health Engagement by Clinical Severity and Symptom Burden

Among participants meeting the ILI definition, 44.13% (95% CI 43.11%-45.16%) reported in-person facility attendance, and 36.07% (95% CI 35.09%-37.07%) reported at least one digital health behavior under the primary channel–based definition. In-person facility attendance was also more frequent among participants with shortness of breath or dyspnea, chest pain, fever, or cough and increased with increasing symptom-count category. These descriptive differences were examined further in the adjusted models. Detailed survey-weighted estimates by ILI status, individual symptoms, and symptom-count category are provided in Table S4 in Multimedia Appendix 2.

### Adjusted Associations With In-Person Facility Attendance

Modified Poisson regression was used to estimate adjusted associations with in-person facility attendance. Results from the overall strict digital health engagement model, symptom-burden model, individual-symptom model, and separate behavior-specific models are shown in Table 3.

Strict digital health engagement was associated with in-person facility attendance during the same ARI episode (aPR 2.12, 95% CI 2.05-2.20). The marginally standardized prevalence of in-person facility attendance was 15.09% (95% CI 14.78%-15.39%) among participants without strict digital health engagement and 31.99% (95% CI 31.16%-32.82%) among those with strict digital health engagement, corresponding to an adjusted prevalence difference of 16.91 percentage points (95% CI 16.01-17.81).

Symptom burden showed the strongest adjusted association with in-person facility attendance. Compared with participants reporting 2 or fewer symptoms, the aPR was 3.31 (95% CI 3.16-3.46) among those with 3 symptoms and 4.69 (95% CI 4.51-4.88) among those with 4 or more symptoms.

In the individual-symptom model, which did not include symptom-count category, in-person facility attendance was positively associated with cough (aPR 2.13, 95% CI 2.05-2.21), fever (aPR 1.73, 95% CI 1.67-1.79), sore throat (aPR 1.66, 95% CI 1.61-1.72), nasal congestion or rhinorrhea (aPR 1.43, 95% CI 1.39-1.48), expectoration (aPR 1.37, 95% CI 1.32-1.42), and shortness of breath or dyspnea (aPR 1.25, 95% CI 1.16-1.34). The CIs for chest pain and nausea or vomiting included the null value.

In the demographic and symptom-burden model, participants living in low-gross domestic product areas had a higher prevalence of in-person facility attendance than those living in high-gross domestic product areas (aPR 1.19, 95% CI 1.15-1.24), whereas those living in middle-gross domestic product areas had a lower prevalence (aPR 0.73, 95% CI 0.70-0.75). Compared with children younger than 5 years, the aPRs were 0.74 (95% CI 0.70-0.79) for participants aged 5-17 years, 0.60 (95% CI 0.56-0.64) for those aged 18-59 years, and 0.69 (95% CI 0.65-0.74) for those aged 60 years or older. Female participants had a slightly higher prevalence than male participants (aPR 1.07, 95% CI 1.04-1.11), and rural residents had a higher prevalence than urban residents (aPR 1.13, 95% CI 1.10-1.17). The association with smartphone ownership was small (aPR 1.05, 95% CI 1.00-1.10). Complete coefficients from the demographic and symptom-burden model are provided in Table S5 in Multimedia Appendix 2.

In separate episode-level models adjusted for demographic characteristics and symptom burden, each measured behavior was positively associated with in-person facility attendance. The aPRs were 1.95 (95% CI 1.89-2.02) for online health information seeking, 2.06 (95% CI 1.98-2.15) for AI-assisted consultation, 2.49 (95% CI 2.39-2.59) for telemedicine, 1.96 (95% CI 1.89-2.03) for offline medication purchase, 1.54 (95% CI 1.47-1.61) for online medication purchase, 2.13 (95% CI 2.04-2.23) for wearable device–based self-monitoring, 1.92 (95% CI 1.79-2.05) for self-administered rapid antigen testing accessed through a digital channel, and 2.88 (95% CI 2.71-3.05) for at-home pathogen specimen collection.

These estimates describe cross-sectional associations within the same illness episode and should not be interpreted as evidence that the behaviors caused, delayed, prevented, or substituted for in-person facility attendance.

**Table 3:** Modified Poisson regression analyses of factors associated with in-person facility attendance among participants with acute respiratory infection symptoms in an 11-city cross-sectional survey in China, January 13-27, 2025^a^.

Variable				Prevalence ratio (95% CI)
**Overall strict digital health engagement and symptom burden**				
	**Overall strict digital health engagement**			
		Crude prevalence ratio	2.90 (2.81-2.99)	
		Adjusted prevalence ratio	2.12 (2.05-2.20)	
	**Symptom count (reference ≤2 symptoms)**			
		3 symptoms	3.31 (3.16-3.46)	
		≥4 symptoms	4.69 (4.51-4.88)	
**Individual-symptom model**				
	Fever		1.73 (1.67-1.79)	
	Sore throat		1.66 (1.61-1.72)	
	Headache		1.10 (1.05-1.14)	
	Generalized body aches		1.16 (1.11-1.21)	
	Fatigue		1.03 (0.98-1.07)	
	Nasal congestion/rhinorrhea		1.43 (1.39-1.48)	
	Tonsillar swelling		1.08 (1.03-1.14)	
	Nausea/vomiting		0.95 (0.89-1.00)	
	Cough		2.13 (2.05-2.21)	
	Sputum production		1.37 (1.32-1.42)	
	Abdominal pain/diarrhea		0.93 (0.87-0.99)	
	Conjunctival congestion		0.70 (0.62-0.80)	
	Change in smell/taste		1.02 (0.95-1.10)	
	Chills		1.00 (0.95-1.06)	
	Shortness of breath/difficulty breathing		1.25 (1.16-1.34)	
	Chest pain		1.06 (0.96-1.17)	
**Separate behavior-specific models**				
	Online health information seeking		1.95 (1.89-2.02)	
	AI-assisted consultation		2.06 (1.98-2.15)	
	Telemedicine		2.49 (2.39-2.59)	
	Offline medication purchase		1.96 (1.89-2.02)	
	Online medication purchase		1.54 (1.47-1.61)	
	Wearable monitoring		2.13 (2.04-2.23)	
	Digitally accessed self-administered rapid antigen testing		1.92 (1.79-2.05)	
	At-home pathogen specimen collection		2.88 (2.71-3.05)	

^a^Modified Poisson regression with a log link, poststratification weights, and individual-level robust (sandwich) variance estimation was used. Overall strict digital health engagement and symptom burden were evaluated in separate models. The overall strict digital health engagement model adjusted for age group, sex, urban-rural residence, regional economic level, smartphone ownership, and symptom-count category; the symptom-burden model adjusted for the demographic covariates. The individual-symptom model included the demographic covariates and all prespecified individual symptom indicators but excluded symptom-count category. Each behavior was evaluated in a separate model adjusted for the demographic covariates and symptom-count category; behaviors were not mutually adjusted. All models used the 68,650 participants reporting recent acute respiratory infection symptoms. Estimates represent cross-sectional associations and should not be interpreted causally.

### Reported Behavioral Sequences and Empirical Transition Patterns

Of the 68,650 participants with recent ARI symptoms, 26,494 (38.59%) reported at least one dated representative action and contributed a nonempty sequence. A total of 6631 participants, corresponding to 9.66% (6631/68,650) of all participants with ARI and 25.03% (6631/26,494) of those with nonempty sequences, contributed more than 1 transition, including the terminal transition appended to each sequence. Multiple actions on the same reported day occurred in 9987 (14.55%) participants.

Among participants with nonempty sequences (n=26,494), 19,863 (74.97%) contributed 1 transition, 5635 (21.27%) contributed 2 transitions, 850 (3.21%) contributed 3 transitions, 131 (0.49%) contributed 4 transitions, 12 (0.05%) contributed 5 transitions, and 3 (0.01%) contributed 6 transitions.

The estimated probability of no subsequent reported action was 0.78 (95% CI 0.77-0.79) following offline medication purchase, 0.74 (95% CI 0.72-0.76) following online medication purchase, 0.67 (95% CI 0.65-0.68) following online health information seeking, 0.66 (95% CI 0.64-0.68) following AI-assisted consultation, and 0.66 (95% CI 0.64-0.69) following telemedicine.

The probability that the next reported state was in-person facility attendance was 0.19 (95% CI 0.16-0.21) following self-administered rapid antigen testing, 0.15 (95% CI 0.14-0.16) following offline medication purchase, 0.15 (95% CI 0.14-0.16) following online medication purchase, 0.14 (95% CI 0.12-0.15) following telemedicine, 0.13 (95% CI 0.12-0.14) following online health information seeking, and 0.13 (95% CI 0.12-0.15) following AI-assisted consultation. The complete empirical adjacent-transition matrix is provided in Table S6 in Multimedia Appendix 2.

Among participants with nonempty sequences, the most frequently reported behavioral sequence was in-person facility attendance followed by no subsequent reported action (8352/26,494, 31.52%), followed by offline medication purchase and no subsequent reported action (6058/26,494, 22.87%), online medication purchase and no subsequent reported action (1601/26,494, 6.04%), online health information seeking and no subsequent reported action (1369/26,494, 5.17%), and offline medication purchase followed by in-person facility attendance and then no subsequent reported action (1202/26,494, 4.54%; Figure 3). Additional details on sequence inclusion, participant-level transition contributions, and the ranked complete sequences are provided in Table S7 in Multimedia Appendix 2. Detailed sensitivity analyses for digital health classification and model specification are provided in Table S8 in Multimedia Appendix 2.

**Figure 3:**
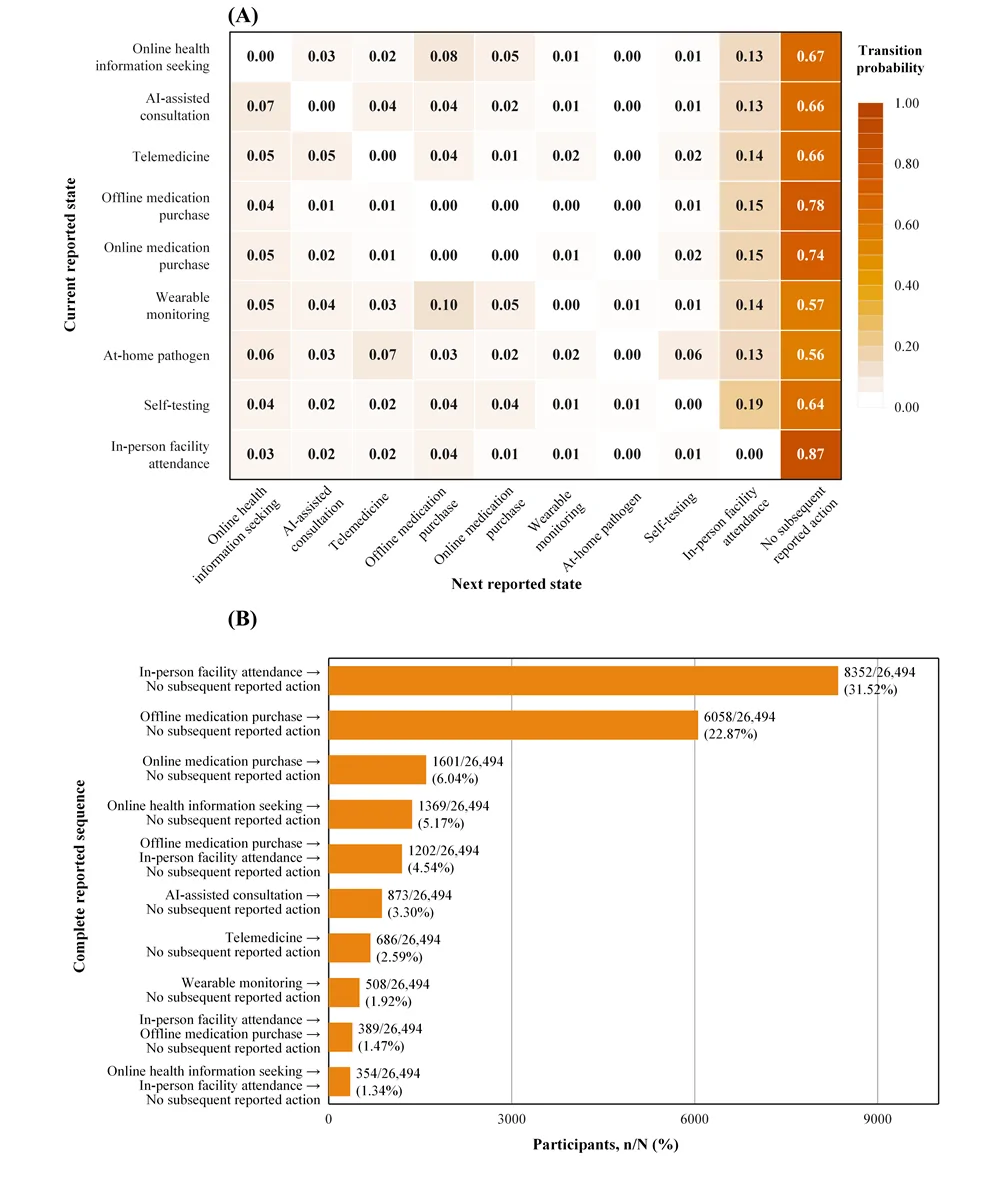
Empirical adjacent transitions and frequently reported complete behavioral sequences after acute respiratory infection symptom onset. Panel A presents the empirical probability that one reported action was followed by another adjacent reported action after applying the prespecified same-day representative-state rule. A terminal "no subsequent reported action" state was appended to each participant with at least one action with usable timing information. Confidence intervals were obtained using 2000 participant-level bootstrap iterations that retained all transitions contributed by each resampled participant. Panel B presents the most frequently reported complete sequences. The analysis describes recalled temporal ordering and does not represent a Markov process or causal pathway.

### Age-Specific Patterns in Facility Attendance and Digital Health Engagement

Age gradients were observed in reported responses among participants with recent ARI symptoms (Figure 4). In-person facility attendance was 25.66% among children younger than 5 years, 19.67% among those aged 5-17 years, 17.61% among adults aged 18-59 years, and 21.80% among adults aged 60 years or older.

**Figure 4:**
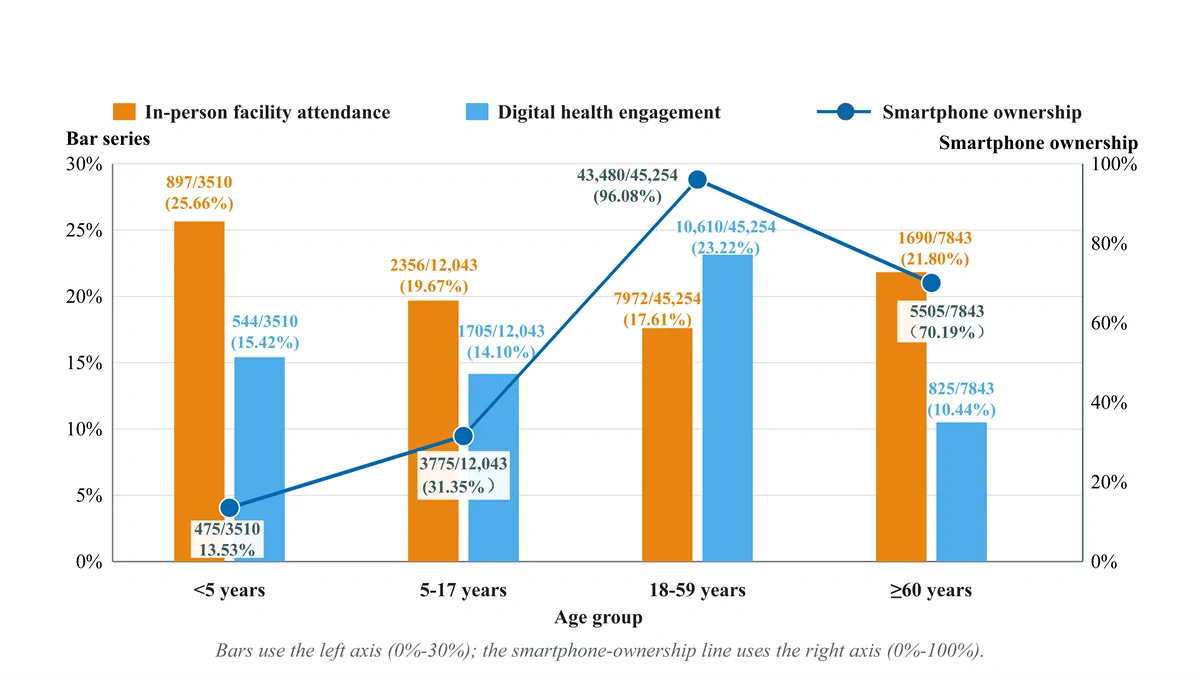
Age-specific survey-weighted proportions of in-person facility attendance, strict digital health engagement, and smartphone ownership among participants reporting recent acute respiratory infection symptoms. Strict digital health engagement follows the primary channel–based definition. The analysis file included only the 68,650 participants reporting recent acute respiratory infection symptoms; therefore, overall age-specific acute respiratory infection prevalence in the complete surveyed population was not independently recalculated or plotted in the final reanalysis.

Under the primary channel–based definition, digital health engagement was most frequent among adults aged 18-59 years (23.22%, 95% CI 22.83%-23.62%) and less frequent among adults aged 60 years or older (10.44%, 95% CI 9.78%-11.14%). The estimates were 15.42% (95% CI 14.26%-16.65%) among children younger than 5 years and 14.10% (95% CI 13.49%-14.74%) among participants aged 5-17 years. Smartphone access also varied markedly across age groups. Detailed behavioral estimates using the alternative adult age categorization are presented in Table S9 in Multimedia Appendix 2. The corresponding modified Poisson regression results using the alternative adult age categorization are presented in Table S10 in Multimedia Appendix 2.

These findings indicate an age-related digital participation gap. Digital data sources derived from these behaviors may generate signals more frequently among some age groups than others; however, direct assessment of the representativeness of specific digital data streams was outside the scope of this study.

## Discussion

### Summary of Key Findings

Most reported ARI episodes did not involve in-person facility attendance and may therefore be underrepresented in routine facility-based surveillance [4-7]. The reported digital health behaviors identify candidate data streams that could potentially complement facility-based surveillance, although their accessibility, validity, timeliness, and incremental surveillance value require direct evaluation [3,8-10,13,14]. The sequence analysis identified several common behavioral pathways after symptom onset, including pathways centered on offline medication purchase, suggesting that nondigital, out-of-facility data may also have surveillance value [15,16]. Digital health engagement was substantially lower among older adults, indicating an important digital divide and a risk that surveillance systems relying heavily on digital traces may underrepresent this population [17-19].

### Interpretation and Comparison

Our findings support prior evidence that responses to respiratory symptoms are heterogeneous and that many symptomatic individuals do not attend a health care facility [4-7,13]. This analysis extends that work by distinguishing 2 dimensions of an ARI episode: whether the individual attended a health care facility in person and whether the episode involved a digitally mediated health-related action. These dimensions could occur independently or together, while specific nondigital, out-of-facility actions were described separately.

Digital health engagement occurred across multiple reported response patterns rather than following a single uniform pathway. Individuals could combine digital and nondigital actions within the same episode, and some reported sequences did not include in-person facility attendance during the reported illness period. The adjacent-transition analysis describes recalled ordering only and does not establish causal or mechanistic pathways.

Some episodes involving digital health engagement did not include subsequent reported in-person facility attendance during the reported illness period. These findings show that digital health engagement can occur outside face-to-face facility-based care, but the cross-sectional data cannot determine whether digital health behaviors substituted for in-person facility attendance, preceded or delayed attendance, reflected milder illness, or were associated with unmet health care needs. As all behaviors were reported for the same illness episode, the data indicate only whether digital health behaviors, offline pharmacy purchases, and in-person facility attendance occurred during that episode. They cannot establish whether digital health behaviors or offline pharmacy purchases replaced, delayed, or prevented facility-based care.

Contextual factors, including health care accessibility, digital platform availability, and norms regarding symptom management, may influence how digital and facility-based actions coexist. In China, online health information seeking, online medication purchase, telemedicine, and at-home pathogen specimen collection provide multiple channels through which health-related actions can occur outside face-to-face facility visits [11,12].

Digital health behaviors were less frequently reported by older adults and other groups with lower smartphone access [17-19]. Digital data sources derived from these behaviors may therefore generate signals more frequently among some population groups than among others [14,17-19]. However, direct assessment of the representativeness of specific digital data streams was outside the scope of this study.

### Implications and Significance

The primary contribution of this study is to distinguish in-person facility attendance from digital health engagement as 2 separate but overlapping dimensions of an ARI episode. This framework provides a population-level view of which episodes may contribute to facility-derived data, generate signals through digital health behaviors, contribute to both sources, or remain less visible through either measured channel. It therefore offers a clearer behavioral context for interpreting the gap between community ARI burden and routinely observed health care data [3-10,13,14]. Digital health behaviors may provide complementary signals for ARI episodes that do not involve in-person facility attendance. Online health information seeking, AI-assisted consultation, online medication purchase, wearable device–based self-monitoring, and at-home pathogen specimen collection may generate observable signals outside conventional facility-based surveillance [3,8-10,13,14].

Nondigital, out-of-facility behaviors may provide additional information [15,16]. In particular, incorporating aggregated community pharmacy sales or dispensing data could help identify symptom-related activity among individuals who purchased medications offline but did not attend a health care facility or use measured digital health services [15,16]. The reported behavioral sequences may also help clarify where facility-based, digital, and pharmacy-derived signals occur within an illness episode. However, these data sources should be regarded as candidate surveillance inputs rather than validated indicators. Their accessibility, timeliness, population coverage, specificity for ARI, correspondence with ARI incidence, and incremental value beyond established surveillance systems require direct linkage and evaluation [3,14-16]. The findings also have implications for health care planning and equity. ARI-related demand was distributed across information seeking, medication purchase, consultation, testing, monitoring, and face-to-face care, but this pattern should not be interpreted as evidence that digital health reduced total professional resource use or successfully replaced facility-based care. Telemedicine, laboratory testing, and at-home pathogen specimen collection may themselves involve health care professionals and infrastructure.

Moreover, lower digital health engagement among older adults indicates that surveillance systems relying heavily on digital traces may underrepresent populations with lower access, skills, or willingness to use digital services [17-19]. Future multisource surveillance strategies could therefore integrate facility-based, digital, pharmacy-derived, and other relevant nondigital data sources, while applying population-specific calibration and maintaining alternative channels for groups affected by the digital divide [3,14-19].

### Limitations

This study has several limitations. First, ARI symptoms and subsequent actions were retrospectively self-reported over a 14-day recall period and were not clinically or laboratory confirmed, which may have introduced recall and misclassification biases.

Second, the framework measures digital health engagement and in-person facility attendance rather than whether health needs were successfully addressed or the total use of professional health care resources. The absence of in-person facility attendance may reflect mild illness, delayed care, barriers to access, or unmet need. Digital health behaviors also varied in their level of professional involvement, and some digital components of self-testing or subsequent consultation may not have been captured. Moreover, the study assessed the occurrence of digital health behaviors but did not determine whether the resulting data were accessible, timely, representative, or valid for surveillance.

Third, although the study used a multistage stratified random sampling design and poststratification calibration, residual selection bias may remain because of nonparticipation, incomplete questionnaires, site-specific implementation differences, and calibration based on a limited set of demographic variables. Although the additional units were randomly selected from the remaining eligible units using the original procedures, these ad hoc, target-based additions deviated from a fixed pure probability sampling design and may have introduced selection related to local recruitment conditions. Poststratification may not fully correct for selection associated with unmeasured characteristics. The proxy-response indicator was not retained, preventing evaluation of differences between self-reported and proxy-reported responses. Proxy respondents may have differed from participants in their awareness, interpretation, or recall of digital health activities, and some activities may have been facilitated or undertaken by the proxy. Response mode may therefore have confounded observed estimates of digital health engagement, particularly among young children and older or dependent adults; the direction and magnitude of this potential bias are unknown.

Fourth, the same-day action hierarchy imposed an artificial ordering in the sequence analysis. As within-day timing was unavailable, collapsing multiple same-day actions into a single representative state obscured co-occurrence and could alter the distributions of states and transitions. The resulting sequences and adjacent transitions should be interpreted as rule-based descriptive summaries rather than observed clinical pathways or evidence of temporal, causal, or replacement relationships.

### Future Directions

Future research should use prospective or longitudinal designs to clarify the temporal relationships among symptom onset, digital health engagement, offline medication purchase, self-testing, and in-person facility attendance. Linking self-reported behaviors with digital trace data, pharmacy sales or dispensing records, laboratory testing data, and health care utilization records would allow direct evaluation of whether these signals are timely, accessible, and associated with changes in community ARI activity [3,8-10,13-16].

Further studies should assess the clinical and health-service implications of different behavioral pathways, including their associations with delayed care, unmet need, subsequent facility use, clinical outcomes, and costs. Such analyses are needed to determine whether digitally mediated and other out-of-facility responses complement, precede, or partially substitute for face-to-face care in specific population groups.

Future surveillance research should also evaluate whether and how combining facility-derived, digital, pharmacy-derived, and other nondigital data improves population coverage and early signal detection. Particular attention should be given to older adults and other populations with lower digital health engagement, so that integrating digital data does not amplify existing disparities in surveillance visibility or access to health services [17-19].

### Conclusions

Most participants reporting recent ARI symptoms did not report in-person facility attendance during the illness period and would therefore be less likely to contribute encounter-based data to routine facility surveillance. Reported digital and other out-of-facility behaviors identify candidate data streams that could potentially complement facility-based surveillance. These findings describe behaviors reported within the same illness episode and do not establish that digital health use or offline pharmacy purchases replaced facility-based care. However, this study did not assess whether such data were accessible to surveillance systems or whether they validly reflected community ARI activity. The lower prevalence of digital health engagement among older adults further highlights the need to evaluate population coverage and equity before incorporating digital data into multisource surveillance systems.

## Appendix

Multimedia Appendix 1 Survey_Questionnaire.

Multimedia Appendix 2 Supplementary Documents.

## Abbreviations

aPR: adjusted prevalence ratio
ARI: acute respiratory infection
ILI: influenza-like illness
