# Supplementary material for: Digital and Out-of-Facility Care-Seeking Pathways After Acute Respiratory Infection Symptoms in China: Multicity Population-Based Cross-Sectional Study
Source: J Med Internet Res. 2026 Sep 30;28:e96371. doi: 10.2196/96371 (PMC13669710; doi:10.2196/96371)
Supplement: Supplementary file 1 [file jmir_v28i1e96371_app1.docx]

**Multimedia Appendix 1**

**Survey Questionnaire: Behavioral Responses Following Acute Respiratory Infection Symptoms**

尊敬的被访者：

您好！中国医学科学院北京协和医学院群医学及公共卫生学院联合我市（区）疾病预防控制中心诚挚邀请您参加一项关于呼吸道传染病监测的问卷调查。本研究旨在了解人们在出现急性呼吸道感染时的行为（如自我诊疗、信息搜索或购药等），并分析这些行为在传染病监测和预警中的作用，提高我国传染病监测和防控能力。问卷预计需要5分钟左右完成，**所有信息将严格保密**，**仅用于科学研究目的**，**不涉及您的隐私问题**，参与完全自愿，您可随时退出。您的参与对优化疾病监测体系、保护公众健康提有重要的科学意义。如有疑问，请随时联系我们（张老师，zhangting@cams.cn）。如果您已了解并同意参与，请选择“我同意并参与调查”。感谢您的支持！

**我阅读并知晓上述的信息，并自愿参与调查：**□是	□否

**填写说明：**

1. 本问卷**以家庭为单位**进行填写，不涉及任何您个人隐私
2. 我们诚挚希望您或您的**家庭成员在过去14天内出现感冒症状的均参与到本次调查**，若您家庭中某一成员不愿意参与本次调查，可以选择“不参与调查”

**第一部分 家庭信息（不重复出现）**

1.1 您所在地：	省	市	区	街道（乡镇）	社区（村）
1.2 您的家庭人员数（包括您在内的同居人数）：
1.3 过去14天内您和您同居的家人感冒的人数：

□0人 □1人 □2人 □3人 □4人 □5人及以上

1.4 是否包括您本人：

□是 □否

**第二部分 您（家庭成员1、家庭成员2…家庭成员5）的基本信息（按照上述家庭人员数将以下问卷题中的“您”依次替换为“家庭成员1”、“家庭成员2”…“家庭成员5”）**

2.1 您的性别：□男 □女
2.2 您的年龄：
2.3 您是否使用智能手机：□是 □否

**第三部分 行为刻画**

3.1 您过去14天内有无出现以下症状，请对以下症状进行选择：（未出现下述症状的终止调查）

□发热 □咽喉痛 □头痛 □全身酸痛 □乏力 □鼻塞、流涕 □扁桃体肿大

□恶心、呕吐 □咳嗽 □咳痰 □腹痛、腹泻 □眼结膜充血 □嗅（味）觉改变

□畏寒 □气促、呼吸困难 □胸痛 □未出现上述症状

3.2 您目前是否已康复：

□是 □否

3.3 您从**出现症状起到康复**持续了	天。
3.4 您在生病期间是否通过**社交媒体和互联网**（如百度、小红书、微信、抖音、微博、知乎等）搜索疾病相关信息：

□否 □是：

□搜索引擎（百度、必应等）

□文献库（知网、pubmed等）

□卫生健康官方网站（世界卫生组织、疾控中心、卫健委等）

□微信（搜一搜、视频号等）

□小红书

□微博

□抖音

□知乎

□其他：

距生病起第	天

3.5 您在生病期间是否通过**人工智能**（如文心一言、豆包等）了解疾病相关信息：

□否 □是：距生病起第	天

3.6 您在生病期间是否通过**线上或电话问诊**（好大夫、医疗机构小程序等）：

□否 □是：

□电话咨询

□线上医疗平台咨询（好大夫在线、丁香园等）

□医疗机构官方渠道

□其他：

距生病起第	天

3.7 您在生病期间是否有过**缺勤或请假**：

□否 □是：距生病起第	天

3.8 您在生病期间是否有**购药行为**（包括线上、线下）：

□否 □是：

□药店线下购药

□电商平台购药（在电商平台下单购买，一般24-48h内送达）

□外卖平台购药（通过外卖平台下单购买药品，然后由平台合作的药店或药品零售企业进行配送，时效性较网络平台好，一般1小时内即可送达）

距生病起第	天

3.9 选择哪个外卖购药平台：（3.8选择“是”和“外卖购药”）

□美团购药 □京东购药/京东健康 □饿了么购药 □叮当购药 □其他：

3.10 您在生病期间是否使用**可穿戴健康设备自检**（智能手表、医用可穿戴设备等）：

□否 □是：

□智能手环、手表等

□医用可穿戴健康设备（医用动态心电图、医用动态血压计等）

□其他：

距生病起第	天

3.11 您在生病期间是否自行购买**检测试剂**（如新冠抗原检测、流感抗原检测）：

□否 □是：

□药店线下购买

□外卖平台购买

□电商平台购买

距生病起第	天

3.12 您在生病期间是否使用**购买上门采样检测**服务（呼吸道病原体采样检测）：

□否 □是：

□美团居家快检

□京东到家快检

□其他：

距生病起第	天

3.13 您在生病期间是否**自行前往医疗机构就诊**：

□否 □是：距生病起第	天（选择首诊医疗机构类型）

□诊所

□乡镇医院/社区医院

□民营（私立）医院

□二级医院

□三级医院

□校医院

□诊所

□乡镇医院/社区医院

□民营（私立）医院

□儿童专科医院

□妇幼保健院

□二级综合性医院

□三级综合性医院

□不清楚：	医院

3.14 您未前往医疗机构就诊，这是出于何种原因：（3.15和3.16选择“否”）

□自觉病情较轻

□担心个人隐私问题

□担心影响上学

□经济负担重

□担心医院交叉感染

□不知道如何选择合适的医疗机构

□就医不便（挂号难、距离远、流程繁琐、等待时间长等）

□没有时间

**第四部分 意愿调查**

**4.1 未来出现感冒症状时是否会使用外卖平台购药**（外卖平台购药是指通过外卖平台购买药品，并通过外卖配送到家的一种新型购药方式‌。用户可以通过手机APP下单，选择所需药品并在线支付，稍作等待配送员即将药品送达）：（本人问卷和家庭成员问卷【非医疗机构购药行为的途径】均未选择【外卖平台购药】）

□是 □否

**4.2 未来出现感冒症状时是否会购买上门采样检测服务**（‌上门采样检测服务‌是一种便捷的核酸检测服务，用户可以通过在线平台下单，专业人员会携带采样工具上门进行采样，然后将样本送至实验室进行检测，检测内容为甲流、乙流、支原体等多种常见的呼吸道感染病原体。检测完成后，用户可以通过手机等设备随时查看检测结果）：（本人问卷和家庭成员问卷【您在本次生病期间是否购买上门采样检测服务（呼吸道病原体采样检测）】均选择【否】）

□是 □否

**Dear respondent:**

Hello. The School of Population Medicine and Public Health, Chinese Academy of Medical Sciences and Peking Union Medical College, together with the local Center for Disease Control and Prevention in your city (district), sincerely invites you to participate in a questionnaire survey on respiratory infectious disease surveillance. This study aims to understand people's behaviors after developing symptoms of acute respiratory infection, such as self-management, information seeking, and medication purchase, and to examine the role of these behaviors in infectious disease surveillance and early warning. The findings will help improve infectious disease surveillance, prevention, and control in China. The questionnaire is expected to take about 5 minutes to complete. All information will be kept strictly confidential and used only for scientific research. The questionnaire does not collect private information. Participation is entirely voluntary, and you may withdraw at any time. Your participation has important scientific value for optimizing disease surveillance systems and protecting public health. If you have any questions, please contact us at any time (Dr. Zhang, zhangting@cams.cn). If you have understood the above information and agree to participate, please select “I agree and participate in the survey.” Thank you for your support.

I have read and understood the above information and voluntarily agree to participate in this survey: □ Yes	□ No

**Instructions:**

1. This questionnaire is completed at the household level and does not involve any personal privacy.

2. We sincerely invite you and any household members who have had cold-like symptoms in the past 14 days to participate in this survey. If a household member is unwilling to participate, “Do not participate in the survey” may be selected.

# Section 1. Household Information (asked once per household)

1.1 Your location: ______ Province ______ City ______ District/County ______ Subdistrict/Township ______ Community/Village

1.2 Number of household members, including yourself, living together: ______

1.3 Number of people in your household, including yourself, who had cold-like symptoms in the past 14 days:

□ 0 people □ 1 person □ 2 people □ 3 people □ 4 people □ 5 people or more

1.4 Does this include yourself?

□ Yes □ No

# Section 2. Basic Information About You (Household Member 1, Household Member 2, …, Household Member 5)

*According to the number of household members reported above, replace “you” in the following questions with “Household Member 1,” “Household Member 2,” …, “Household Member 5,” as applicable.*

2.1 Your sex: □ Male □ Female

2.2 Your age: ______

2.3 Do you use a smartphone? □ Yes □ No

# Section 3. Behavioral Characterization

3.1 In the past 14 days, have you had any of the following symptoms? Please select all that apply. If none of the following symptoms occurred, terminate the survey.

□ Fever □ Sore throat □ Headache □ Generalized body aches □ Fatigue □ Nasal congestion or runny nose □ Enlarged tonsils

□ Nausea or vomiting □ Cough □ Sputum production □ Abdominal pain or diarrhea □ Conjunctival congestion □ Change in smell or taste

□ Chills □ Shortness of breath or difficulty breathing □ Chest pain □ None of the above symptoms

3.2 Have you recovered now?

□ Yes □ No

3.3 From symptom onset to recovery, your illness lasted ______ days.

3.4 During this illness, did you search for disease-related information through social media or the internet, such as Baidu, Xiaohongshu, WeChat, Douyin, Weibo, or Zhihu?

□ No □ Yes:

□ Search engines (Baidu, Bing, etc.)

□ Literature databases (CNKI, PubMed, etc.)

□ Official health websites (World Health Organization, Centers for Disease Control and Prevention, health commissions, etc.)

□ WeChat (Search, Channels, etc.)

□ Xiaohongshu (RED)

□ Weibo

□ Douyin

□ Zhihu

□ Other: ______

On day ______ after illness onset

3.5 During this illness, did you use artificial intelligence tools, such as ERNIE Bot or Doubao, to obtain disease-related information?

□ No □ Yes: On day ______ after illness onset

3.6 During this illness, did you use online or telephone consultation services, such as Haodf.com or a medical institution mini-program?

□ No □ Yes:

□ Telephone consultation

□ Online medical platform consultation (Haodf.com, DXY, etc.)

□ Official channels of medical institutions

□ Other: ______

On day ______ after illness onset

3.7 During this illness, did you have any absence from work or school or take leave?

□ No □ Yes: On day ______ after illness onset

3.8 During this illness, did you purchase any medications, either online or offline?

□ No □ Yes:

□ Offline purchase at a pharmacy

□ Purchase through an e-commerce platform (placing an order through an e-commerce platform; generally delivered within 24–48 hours)

□ Purchase through an on-demand delivery platform (placing an order through an on-demand delivery platform; medications are then delivered by a partner pharmacy or retail pharmacy enterprise, usually faster than standard online platforms and generally delivered within 1 hour)

On day ______ after illness onset

3.9 Which on-demand delivery platform did you use to purchase medication? (If “Yes” and “on-demand delivery platform” were selected in Q3.8)

□ Meituan Medicine □ JD Medicine/JD Health □ Ele.me Medicine □ Dingdang Medicine Express □ Other: ______

3.10 During this illness, did you use wearable health devices for self-monitoring, such as a smart watch or medical wearable device?

□ No □ Yes:

□ Smart bracelet, smart watch, etc.

□ Medical wearable health device (medical ambulatory electrocardiogram monitor, medical ambulatory blood pressure monitor, etc.)

□ Other: ______

On day ______ after illness onset

3.11 During this illness, did you purchase self-testing kits, such as COVID-19 antigen tests or influenza antigen tests?

□ No □ Yes:

□ Purchased offline at a pharmacy

□ Purchased through an on-demand delivery platform

□ Purchased through an e-commerce platform

On day ______ after illness onset

3.12 During this illness, did you purchase an at-home sample collection and respiratory pathogen testing service?

□ No □ Yes:

□ Meituan Home Rapid Testing

□ JD Daojia Home Rapid Testing

□ Other: ______

On day ______ after illness onset

3.13 During this illness, did you go to a medical facility for an in-person visit on your own initiative?

□ No □ Yes: On day ______ after illness onset (select the type of medical facility for the first visit)

□ Clinic

□ Township hospital/community health service center

□ Private hospital

□ Secondary hospital

□ Tertiary hospital

□ School infirmary/hospital

□ Pediatric specialty hospital

□ Maternal and child health hospital

□ Secondary general hospital

□ Tertiary general hospital

□ Not sure: ______ hospital

3.14 If you did not go to a medical facility, what were the reasons?

□ Felt that the illness was mild

□ Concern about personal privacy

□ Concern about affecting school attendance

□ Heavy financial burden

□ Concern about cross-infection in the hospital

□ Did not know how to choose an appropriate medical facility

□ Inconvenient to seek care, such as difficulty making appointments, long distance, complicated procedures, or long waiting time

□ No time

# Section 4. Intention Survey

4.1 If you develop cold-like symptoms in the future, would you use an on-demand delivery platform to purchase medication?

*An on-demand delivery platform medication purchase refers to a new way of buying medications through an on-demand delivery platform and having them delivered to your home. Users can place an order through a mobile app, select the required medications, pay online, and wait for the delivery worker to deliver the medications.*

*Logic condition: asked only if neither the respondent nor household-member questionnaires selected “on-demand delivery platform” as a route for non-facility medication purchase.*

□ Yes □ No

4.2 If you develop cold-like symptoms in the future, would you purchase an at-home sample collection and respiratory pathogen testing service?

*An at-home sample collection and testing service is a convenient nucleic acid/pathogen testing service. Users can place an order through an online platform, and trained staff will bring sampling tools to the user's home to collect a specimen and then send the specimen to a laboratory for testing. The test covers common respiratory infection pathogens, including influenza A, influenza B, and Mycoplasma. After testing is completed, users can view the results at any time on a mobile phone or other device.*

*Logic condition: asked only if both the respondent and household-member questionnaires selected “No” for purchasing an at-home sample collection and testing service during this illness.*

□ Yes □ No
