# Supplementary material for: Digital and Out-of-Facility Care-Seeking Pathways After Acute Respiratory Infection Symptoms in China: Multicity Population-Based Cross-Sectional Study
Source: J Med Internet Res. 2026 Sep 30;28:e96371. doi: 10.2196/96371 (PMC13669710; doi:10.2196/96371)
Supplement: Supplementary file 2 [file jmir_v28i1e96371_app2.docx]

### ****Multimedia Appendix 2****

**Digital and Out-of-Facility Care-Seeking Pathways After Acute Respiratory Infection Symptoms in China: A Multicity Population-Based Cross-Sectional Study**

**Contents**

Methods S1. Reconstruction of Reported Behavioral Sequences	2

Table S1. City-level survey initiation, analytic inclusion, and exclusion in the 11-city ARI survey, January 13–27, 2025.	3

Table S2. Acute respiratory infection prevalence by demographic and geographic characteristics.	4

Table S3. Survey-weighted prevalence of in-person facility attendance and out-of-facility behaviors by baseline demographic characteristics.	6

Table S4. Survey-weighted prevalence of in-person facility attendance and out-of-facility behaviors by influenza-like illness status, symptoms, and symptom burden.	8

Table S5. Full coefficients from the demographic and symptom-burden modified Poisson model for in-person facility attendance.	10

Table S6. Complete empirical adjacent-transition matrix among participants with nonempty behavioral sequences.	11

Table S7. Sequence inclusion, participant-level transition contributions, and most frequently reported complete behavioral sequences.	15

Table S8. Sensitivity analyses for digital health classification and model specification.	16

Table S9. Survey-weighted prevalence of digital health engagement, in-person facility attendance, and specific behaviors using an alternative adult age categorization.	19

Table S10. Modified Poisson regression using the alternative adult age categorization.	21

## **Methods S1. Reconstruction of Reported Behavioral Sequences**

**S1.1 Questionnaire Structure and Temporal Ordering**

Participants retrospectively reported the actions undertaken after acute respiratory infection symptom onset and the calendar date of each selected action. Each behavioral category could be selected and dated only once within an illness episode. Sequences were therefore reconstructed from reported dates relative to symptom onset; repeated occurrences of the same behavior on different dates could not be recorded.

**S1.2 Handling of Multiple Actions Reported on the Same Day**

When multiple actions were reported on the same calendar day, one representative state was retained using the following prespecified priority:

1. In-person facility attendance

2. Online health information seeking

3. AI-assisted consultation

4. Telemedicine

5. Offline medication purchase

6. Online medication purchase

7. Wearable device–based self-monitoring

8. Self-administered rapid antigen testing

9. At-home pathogen specimen collection

This priority rule was used only to generate an analyzable day-level sequence. It does not establish the true within-day order of actions and does not imply clinical importance, causality, or treatment escalation.

**S1.3 Eligibility for Sequence Analysis**

Episodes were included in the sequence analysis when at least one dated representative action could be identified. One representative state was retained for each reported calendar day, and a terminal “No subsequent reported action” state was appended to every nonempty sequence. Episodes with no dated action or nonorderable timing information were excluded from sequence-based analyses but retained in descriptive analyses. Thus, a single dated action formed a nonempty sequence consisting of that action followed by the terminal state.

**S1.4 Estimation and Interpretation of Behavioral Transitions**

Only transitions between adjacent dated representative states were counted. For each origin state, the empirical adjacent-transition probability was the observed number of transitions to a given next state divided by the total number of transitions from that origin state. A terminal “No subsequent reported action” state was appended to every nonempty sequence. Confidence intervals were calculated using 2000 participant-level bootstrap iterations; participants were resampled and all transitions contributed by each selected participant were retained together. The analysis describes recalled temporal ordering and does not represent a generative Markov process, causal pathway, or mechanistic transition rate.

“No subsequent reported action” indicates only that no later dated action was recorded in the questionnaire. It does not indicate symptom resolution, successful self-management, deliberate watchful waiting, or absence of subsequent healthcare use.

## **Table S1. City-level survey initiation, analytic inclusion, and exclusion in the 11-city ARI survey, January 13–27, 2025.**

| **City** | **Initiated, n** | **Included in analytic sample, n** | **Excluded, n** | **Analytic inclusion proportion, %** |
| --- | --- | --- | --- | --- |
| Baiyin | 14,367 | 13,344 | 1,023 | 92.90 |
| Chengdu | 25,501 | 23,004 | 2,497 | 90.20 |
| Dalian | 51,091 | 46,430 | 4,661 | 90.90 |
| Hangzhou | 18,152 | 16,507 | 1,645 | 90.90 |
| Shanghai | 16,801 | 15,737 | 1,064 | 93.70 |
| Shenyang | 61,398 | 56,679 | 4,719 | 92.30 |
| Suining | 21,510 | 19,560 | 1,950 | 90.90 |
| Weifang | 19,351 | 17,464 | 1,887 | 90.20 |
| Xiangtan | 8,836 | 8,571 | 265 | 97.00 |
| Yichang | 16,546 | 15,195 | 1,351 | 91.80 |
| Chongqing | 15,223 | 15,039 | 184 | 98.80 |
| Total | 268,776 | 247,530 | 21,246 | 92.10 |

Note. Initiated refers to residents who consented and started the questionnaire. Excluded records were incomplete, did not meet eligibility criteria, or failed prespecified quality-control checks. The overall 92.10% is the analytic inclusion proportion among those who initiated the survey; it is not a survey response rate.

## **Table S2. Acute respiratory infection prevalence by demographic and geographic characteristics.**

| **Variable** | **<5 years, n/N (%; 95% CI)** | **5–17 years, n/N (%; 95% CI)** | **18–59 years, n/N (%; 95% CI)** | **≥60 years, n/N (%; 95% CI)** | **Crude prevalence, n/N (%; 95% CI)** | **Weighted prevalence, % (95% CI)** |
| --- | --- | --- | --- | --- | --- | --- |
| Overall | 3,510/9,365 (37.48; 36.50–38.46) | 12,043/42,448 (28.37; 27.94–28.80) | 45,254/163,785 (27.63; 27.41–27.85) | 7,843/31,932 (24.56; 24.09–25.03) | 68,650/247,530 (27.73; 27.56–27.91) | 27.40 (27.22–27.59) |
| **Sex** |  |  |  |  |  |  |
| Male | 1,862/4,876 (38.19; 36.82–39.55) | 6,287/21,654 (29.03; 28.43–29.64) | 18,226/73,017 (24.96; 24.65–25.28) | 3,530/14,825 (23.81; 23.13–24.50) | 29,905/114,372 (26.15; 25.89–26.40) | 26.00 (25.74–26.27) |
| Female | 1,648/4,489 (36.71; 35.30–38.12) | 5,756/20,794 (27.68; 27.07–28.29) | 27,028/90,768 (29.78; 29.48–30.07) | 4,313/17,107 (25.21; 24.56–25.86) | 38,745/133,158 (29.10; 28.85–29.34) | 28.78 (28.53–29.03) |
| **Smartphone** |  |  |  |  |  |  |
| Yes | 470/1,190 (39.50; 36.72–42.27) | 3,771/14,323 (26.33; 25.61–27.05) | 43,494/157,911 (27.54; 27.32–27.76) | 5,526/23,006 (24.02; 23.47–24.57) | 53,261/196,430 (27.11; 26.92–27.31) | 26.75 (26.54–26.95) |
| No | 3,040/8,175 (37.19; 36.14–38.23) | 8,272/28,125 (29.41; 28.88–29.94) | 1,760/5,874 (29.96; 28.79–31.13) | 2,317/8,926 (25.96; 25.05–26.87) | 15,389/51,100 (30.12; 29.72–30.51) | 29.81 (29.39–30.22) |
| **City** |  |  |  |  |  |  |
| Chongqing | 306/852 (35.92; 32.69–39.14) | 553/1,941 (28.49; 26.48–30.50) | 3,472/9,223 (37.65; 36.66–38.63) | 1,306/3,023 (43.20; 41.44–44.97) | 5,637/15,039 (37.48; 36.71–38.26) | 38.23 (37.42–39.04) |
| Xiangtan | 152/346 (43.93; 38.70–49.16) | 434/1,356 (32.01; 29.52–34.49) | 1,593/5,076 (31.38; 30.11–32.66) | 554/1,793 (30.90; 28.76–33.04) | 2,733/8,571 (31.89; 30.90–32.87) | 32.04 (31.01–33.08) |
| Baiyin | 351/845 (41.54; 38.22–44.86) | 1,164/2,837 (41.03; 39.22–42.84) | 2,599/7,993 (32.52; 31.49–33.54) | 536/1,669 (32.12; 29.87–34.36) | 4,650/13,344 (34.85; 34.04–35.66) | 34.40 (33.57–35.24) |
| Weifang | 279/717 (38.91; 35.34–42.48) | 1,032/2,755 (37.46; 35.65–39.27) | 4,548/12,253 (37.12; 36.26–37.97) | 559/1,739 (32.14; 29.95–34.34) | 6,418/17,464 (36.75; 36.03–37.46) | 36.24 (35.51–36.98) |
| Yichang | 229/563 (40.67; 36.62–44.73) | 528/1,560 (33.85; 31.50–36.19) | 3,518/11,268 (31.22; 30.37–32.08) | 464/1,804 (25.72; 23.70–27.74) | 4,739/15,195 (31.19; 30.45–31.92) | 30.69 (29.94–31.45) |
| Dalian | 451/1,394 (32.35; 29.90–34.81) | 1,921/6,284 (30.57; 29.43–31.71) | 9,378/32,853 (28.55; 28.06–29.03) | 1,395/5,899 (23.65; 22.56–24.73) | 13,145/46,430 (28.31; 27.90–28.72) | 27.69 (27.27–28.11) |
| Shanghai | 174/474 (36.71; 32.37–41.05) | 865/2,534 (34.14; 32.29–35.98) | 2,691/10,318 (26.08; 25.23–26.93) | 425/2,411 (17.63; 16.11–19.15) | 4,155/15,737 (26.40; 25.71–27.09) | 25.29 (24.59–26.00) |
| Chengdu | 415/939 (44.20; 41.02–47.37) | 965/3,148 (30.65; 29.04–32.27) | 4,541/16,071 (28.26; 27.56–28.95) | 641/2,846 (22.52; 20.99–24.06) | 6,562/23,004 (28.53; 27.94–29.11) | 27.96 (27.37–28.57) |
| Suining | 226/608 (37.17; 33.33–41.01) | 965/3,799 (25.40; 24.02–26.79) | 2,700/12,067 (22.38; 21.63–23.12) | 574/3,086 (18.60; 17.23–19.97) | 4,465/19,560 (22.83; 22.24–23.42) | 22.26 (21.66–22.87) |
| Shenyang | 811/2,233 (36.32; 34.32–38.31) | 2,501/10,968 (22.80; 22.02–23.59) | 8,649/37,562 (23.03; 22.60–23.45) | 1,198/5,916 (20.25; 19.23–21.27) | 13,159/56,679 (23.22; 22.87–23.56) | 22.90 (22.54–23.26) |
| Hangzhou | 116/394 (29.44; 24.94–33.94) | 1,115/5,266 (21.17; 20.07–22.28) | 1,565/9,101 (17.20; 16.42–17.97) | 191/1,746 (10.94; 9.48–12.40) | 2,987/16,507 (18.10; 17.51–18.68) | 17.39 (16.80–17.99) |

**Note.** ARI indicates acute respiratory infection; CI, confidence interval. Crude 95% CIs are unweighted Wald intervals. Weighted point estimates use poststratification weights calibrated to age group, sex, and urban-rural residence. Weighted 95% CIs are Wilson score intervals based on the Kish effective sample size derived from the poststratification weights within each subgroup.

## **Table S3. Survey-weighted prevalence of in-person facility attendance and out-of-facility behaviors by baseline demographic characteristics.**

| **Characteristic** | **Unweighted n** | **In-person facility attendance, weighted % (95% CI)** | **Offline medication purchase, weighted % (95% CI)** | **Digital health engagement under the primary channel-based definition, weighted % (95% CI)** |
| --- | --- | --- | --- | --- |
| **City** |  |  |  |  |
| Baiyin | 4,650 | 25.02% (95% CI 23.75–26.33) | 24.74% (95% CI 23.48–26.04) | 17.21% (95% CI 16.11–18.36) |
| Chengdu | 6,562 | 22.69% (95% CI 21.66–23.75) | 19.97% (95% CI 18.99–20.98) | 18.04% (95% CI 17.10–19.02) |
| Dalian | 13,145 | 14.55% (95% CI 13.94–15.18) | 21.26% (95% CI 20.55–21.99) | 22.02% (95% CI 21.30–22.76) |
| Hangzhou | 2,987 | 17.18% (95% CI 15.84–18.61) | 11.44% (95% CI 10.32–12.66) | 16.45% (95% CI 15.13–17.86) |
| Shanghai | 4,155 | 25.49% (95% CI 24.15–26.88) | 7.82% (95% CI 7.02–8.71) | 29.13% (95% CI 27.73–30.58) |
| Shenyang | 13,159 | 11.96% (95% CI 11.40–12.54) | 17.12% (95% CI 16.47–17.79) | 20.59% (95% CI 19.89–21.31) |
| Suining | 4,465 | 27.59% (95% CI 26.25–28.97) | 18.86% (95% CI 17.70–20.08) | 14.82% (95% CI 13.77–15.93) |
| Weifang | 6,418 | 20.49% (95% CI 19.49–21.52) | 18.89% (95% CI 17.93–19.89) | 18.81% (95% CI 17.85–19.81) |
| Xiangtan | 2,733 | 26.15% (95% CI 24.47–27.90) | 29.30% (95% CI 27.55–31.11) | 15.27% (95% CI 13.91–16.73) |
| Yichang | 4,739 | 18.80% (95% CI 17.68–19.97) | 26.11% (95% CI 24.85–27.42) | 17.62% (95% CI 16.53–18.76) |
| Chongqing | 5,637 | 21.45% (95% CI 20.35–22.59) | 20.80% (95% CI 19.71–21.93) | 11.62% (95% CI 10.77–12.53) |
| **Sex** |  |  |  |  |
| Male | 29,905 | 18.56% (95% CI 18.11–19.02) | 19.34% (95% CI 18.88–19.80) | 17.13% (95% CI 16.69–17.57) |
| Female | 38,745 | 19.73% (95% CI 19.32–20.14) | 19.95% (95% CI 19.54–20.36) | 20.55% (95% CI 20.14–20.97) |
| **Urban or rural residence** |  |  |  |  |
| Urban | 40,806 | 17.20% (95% CI 16.82–17.58) | 17.89% (95% CI 17.51–18.27) | 20.43% (95% CI 20.03–20.83) |
| Rural | 27,844 | 21.68% (95% CI 21.19–22.19) | 21.90% (95% CI 21.40–22.41) | 17.06% (95% CI 16.61–17.52) |
| **Regional GDP level** |  |  |  |  |
| High GDP | 19,341 | 22.12% (95% CI 21.52–22.73) | 16.41% (95% CI 15.87–16.96) | 18.15% (95% CI 17.59–18.72) |
| Middle GDP | 37,461 | 15.23% (95% CI 14.86–15.61) | 20.06% (95% CI 19.65–20.48) | 20.40% (95% CI 19.98–20.82) |
| Low GDP | 11,848 | 26.25% (95% CI 25.43–27.08) | 23.67% (95% CI 22.88–24.47) | 15.85% (95% CI 15.18–16.54) |
| **Age group** |  |  |  |  |
| <5 years | 3,510 | 25.66% (95% CI 24.23–27.13) | 15.71% (95% CI 14.54–16.96) | 15.42% (95% CI 14.26–16.65) |
| 5–17 years | 12,043 | 19.67% (95% CI 18.97–20.39) | 16.61% (95% CI 15.95–17.29) | 14.10% (95% CI 13.49–14.74) |
| 18–59 years | 45,254 | 17.61% (95% CI 17.26–17.96) | 20.71% (95% CI 20.34–21.09) | 23.22% (95% CI 22.83–23.62) |
| ≥60 years | 7,843 | 21.80% (95% CI 20.90–22.73) | 19.97% (95% CI 19.10–20.88) | 10.44% (95% CI 9.78–11.14) |

**Note.** Values are unweighted numbers and survey-weighted proportions with 95% CIs. Digital health engagement used the primary channel-based definition. Self-administered rapid antigen testing contributed only when the test kit was obtained through an e-commerce or on-demand delivery platform; at-home pathogen specimen collection was classified as digitally arranged. Weighted 95% CIs are Wilson score intervals based on the Kish effective sample size derived from poststratification weights calibrated to age group, sex, and urban-rural residence within each subgroup. GDP indicates gross domestic product.

## **Table S4. Survey-weighted prevalence of in-person facility attendance and out-of-facility behaviors by influenza-like illness status, symptoms, and symptom burden.**

| **Clinical characteristic** | **Unweighted n** | **In-person facility attendance, weighted % (95% CI)** | **Offline medication purchase, weighted % (95% CI)** | **Digital health engagement under the primary channel-based definition, weighted % (95% CI)** |
| --- | --- | --- | --- | --- |
| **Overall** |  |  |  |  |
| Participants with recent ARI symptoms | 68,650 | 19.18% (95% CI 18.87–19.48) | 19.66% (95% CI 19.36–19.97) | 18.94% (95% CI 18.64–19.24) |
| **ILI status** |  |  |  |  |
| Non-ILI | 58,988 | 15.09% (95% CI 14.79–15.39) | 16.94% (95% CI 16.63–17.26) | 16.13% (95% CI 15.83–16.44) |
| ILI | 9,662 | 44.13% (95% CI 43.11–45.16) | 36.25% (95% CI 35.27–37.25) | 36.07% (95% CI 35.09–37.07) |
| **Individual symptoms** |  |  |  |  |
| Fever | 19,148 | 31.44% (95% CI 30.76–32.12) | 27.07% (95% CI 26.43–27.73) | 28.63% (95% CI 27.97–29.29) |
| Sore throat | 27,222 | 27.56% (95% CI 27.01–28.11) | 29.03% (95% CI 28.47–29.59) | 29.93% (95% CI 29.37–30.49) |
| Headache | 15,889 | 27.41% (95% CI 26.70–28.13) | 27.37% (95% CI 26.66–28.09) | 29.29% (95% CI 28.56–30.03) |
| Generalized body aches | 14,724 | 29.59% (95% CI 28.83–30.36) | 30.01% (95% CI 29.25–30.78) | 30.16% (95% CI 29.39–30.93) |
| Fatigue | 14,460 | 27.66% (95% CI 26.91–28.42) | 28.43% (95% CI 27.68–29.20) | 30.33% (95% CI 29.56–31.12) |
| Nasal congestion/rhinorrhea | 25,980 | 26.49% (95% CI 25.94–27.05) | 28.07% (95% CI 27.50–28.63) | 26.21% (95% CI 25.66–26.76) |
| Tonsillar swelling | 3,499 | 37.25% (95% CI 35.61–38.91) | 33.12% (95% CI 31.53–34.74) | 37.86% (95% CI 36.22–39.53) |
| Nausea/vomiting | 4,664 | 25.85% (95% CI 24.58–27.17) | 23.46% (95% CI 22.23–24.73) | 28.92% (95% CI 27.60–30.28) |
| Cough | 32,185 | 28.85% (95% CI 28.34–29.36) | 28.46% (95% CI 27.95–28.97) | 25.35% (95% CI 24.87–25.85) |
| Sputum production | 14,610 | 36.22% (95% CI 35.42–37.04) | 33.78% (95% CI 32.98–34.58) | 31.25% (95% CI 30.48–32.04) |
| Abdominal pain/diarrhea | 4,399 | 21.31% (95% CI 20.10–22.58) | 21.20% (95% CI 19.99–22.46) | 25.48% (95% CI 24.19–26.83) |
| Conjunctival congestion | 754 | 28.34% (95% CI 25.13–31.78) | 25.64% (95% CI 22.55–28.99) | 35.38% (95% CI 31.93–38.98) |
| Change in smell/taste | 1,747 | 40.73% (95% CI 38.38–43.13) | 40.90% (95% CI 38.55–43.31) | 41.56% (95% CI 39.20–43.97) |
| Chills | 4,273 | 34.40% (95% CI 32.94–35.89) | 32.44% (95% CI 31.00–33.91) | 34.72% (95% CI 33.25–36.21) |
| Shortness of breath/difficulty breathing | 1,887 | 46.84% (95% CI 44.49–49.20) | 39.39% (95% CI 37.11–41.72) | 40.76% (95% CI 38.46–43.10) |
| Chest pain | 1,139 | 40.49% (95% CI 37.58–43.47) | 37.41% (95% CI 34.55–40.36) | 45.31% (95% CI 42.33–48.31) |
| **Symptom-count category** |  |  |  |  |
| ≤2 symptoms | 40,554 | 8.34% (95% CI 8.06–8.62) | 9.79% (95% CI 9.50–10.09) | 9.21% (95% CI 8.92–9.51) |
| 3 symptoms | 11,020 | 28.46% (95% CI 27.59–29.34) | 29.03% (95% CI 28.16–29.91) | 26.40% (95% CI 25.56–27.26) |
| ≥4 symptoms | 17,076 | 38.70% (95% CI 37.95–39.46) | 36.83% (95% CI 36.09–37.58) | 37.02% (95% CI 36.28–37.78) |

**Note.** Individual symptom categories are not mutually exclusive; symptom-count categories are mutually exclusive. ILI was defined as fever plus cough or sore throat. Digital health engagement used the primary channel-based definition. Weighted 95% CIs are Wilson score intervals based on the Kish effective sample size derived from poststratification weights calibrated to age group, sex, and urban-rural residence within each subgroup. ARI indicates acute respiratory infection; ILI, influenza-like illness.

## **Table S5. Full coefficients from the demographic and symptom-burden modified Poisson model for in-person facility attendance among participants with acute respiratory infection symptoms.**

| **Variable/category** | **Reference category** | **aPR** | **95% CI** |
| --- | --- | --- | --- |
| **Age group** | | | |
| 5–17 years | <5 years | 0.74 | 0.70–0.79 |
| 18–59 years | <5 years | 0.60 | 0.56–0.64 |
| ≥60 years | <5 years | 0.69 | 0.65–0.74 |
| **Sex** | | | |
| Female | Male | 1.07 | 1.04–1.11 |
| **Residence** | | | |
| Rural | Urban | 1.13 | 1.10–1.17 |
| **City-level GDP** | | | |
| Middle GDP | High GDP | 0.73 | 0.70–0.75 |
| Low GDP | High GDP | 1.19 | 1.15–1.24 |
| **Smartphone ownership** | | | |
| Owner | Non-owner | 1.05 | 1.00–1.10 |
| **Symptom count** | | | |
| 3 symptoms | ≤2 symptoms | 3.31 | 3.16–3.46 |
| ≥4 symptoms | ≤2 symptoms | 4.69 | 4.51–4.88 |

**Note.** aPR indicates adjusted prevalence ratio; ARI, acute respiratory infection; CI, confidence interval; GDP, gross domestic product. The model used modified Poisson regression with a log link, poststratification weights calibrated to age, sex, and urban-rural residence, and individual-level robust (sandwich) variance estimation. All listed covariates were included simultaneously. Analysis N=68,650. Estimates are cross-sectional associations and should not be interpreted causally.

## **Table S6. Complete empirical adjacent-transition matrix among participants with nonempty behavioral sequences.**

| **From state** | **To state** | **Transition numerator, n** | **Row denominator, N** | **Transition probability** | **Participant-level bootstrap 95% CI** |
| --- | --- | --- | --- | --- | --- |
| In-person facility attendance | Online health information seeking | 387 | 12,915 | 0.03 | 0.03–0.03 |
| In-person facility attendance | AI-assisted consultation | 241 | 12,915 | 0.02 | 0.02–0.02 |
| In-person facility attendance | Telemedicine | 249 | 12,915 | 0.02 | 0.02–0.02 |
| In-person facility attendance | Offline medication purchase | 458 | 12,915 | 0.04 | 0.03–0.04 |
| In-person facility attendance | Online medication purchase | 165 | 12,915 | 0.01 | 0.01–0.01 |
| In-person facility attendance | Wearable device–based self-monitoring | 83 | 12,915 | 0.01 | 0.01–0.01 |
| In-person facility attendance | Self-administered rapid antigen testing | 104 | 12,915 | 0.01 | 0.01–0.01 |
| In-person facility attendance | At-home pathogen specimen collection | 34 | 12,915 | 0.00 | 0.00–0.00 |
| In-person facility attendance | No subsequent reported action | 11,194 | 12,915 | 0.87 | 0.86–0.87 |
| Online health information seeking | In-person facility attendance | 476 | 3,581 | 0.13 | 0.12–0.14 |
| Online health information seeking | AI-assisted consultation | 118 | 3,581 | 0.03 | 0.03–0.04 |
| Online health information seeking | Telemedicine | 77 | 3,581 | 0.02 | 0.02–0.03 |
| Online health information seeking | Offline medication purchase | 270 | 3,581 | 0.08 | 0.07–0.08 |
| Online health information seeking | Online medication purchase | 184 | 3,581 | 0.05 | 0.04–0.06 |
| Online health information seeking | Wearable device–based self-monitoring | 21 | 3,581 | 0.01 | 0.00–0.01 |
| Online health information seeking | Self-administered rapid antigen testing | 35 | 3,581 | 0.01 | 0.01–0.01 |
| Online health information seeking | At-home pathogen specimen collection | 7 | 3,581 | 0.00 | 0.00–0.00 |
| Online health information seeking | No subsequent reported action | 2,393 | 3,581 | 0.67 | 0.65–0.68 |
| AI-assisted consultation | In-person facility attendance | 275 | 2,106 | 0.13 | 0.12–0.15 |
| AI-assisted consultation | Online health information seeking | 151 | 2,106 | 0.07 | 0.06–0.08 |
| AI-assisted consultation | Telemedicine | 77 | 2,106 | 0.04 | 0.03–0.04 |
| AI-assisted consultation | Offline medication purchase | 93 | 2,106 | 0.04 | 0.04–0.05 |
| AI-assisted consultation | Online medication purchase | 51 | 2,106 | 0.02 | 0.02–0.03 |
| AI-assisted consultation | Wearable device–based self-monitoring | 29 | 2,106 | 0.01 | 0.01–0.02 |
| AI-assisted consultation | Self-administered rapid antigen testing | 27 | 2,106 | 0.01 | 0.01–0.02 |
| AI-assisted consultation | At-home pathogen specimen collection | 7 | 2,106 | 0.00 | 0.00–0.01 |
| AI-assisted consultation | No subsequent reported action | 1,396 | 2,106 | 0.66 | 0.64–0.68 |
| Telemedicine | In-person facility attendance | 230 | 1,673 | 0.14 | 0.12–0.15 |
| Telemedicine | Online health information seeking | 83 | 1,673 | 0.05 | 0.04–0.06 |
| Telemedicine | AI-assisted consultation | 82 | 1,673 | 0.05 | 0.04–0.06 |
| Telemedicine | Offline medication purchase | 68 | 1,673 | 0.04 | 0.03–0.05 |
| Telemedicine | Online medication purchase | 21 | 1,673 | 0.01 | 0.01–0.02 |
| Telemedicine | Wearable device–based self-monitoring | 33 | 1,673 | 0.02 | 0.01–0.03 |
| Telemedicine | Self-administered rapid antigen testing | 37 | 1,673 | 0.02 | 0.02–0.03 |
| Telemedicine | At-home pathogen specimen collection | 8 | 1,673 | 0.00 | 0.00–0.01 |
| Telemedicine | No subsequent reported action | 1,111 | 1,673 | 0.66 | 0.64–0.69 |
| Offline medication purchase | In-person facility attendance | 1,344 | 8,919 | 0.15 | 0.14–0.16 |
| Offline medication purchase | Online health information seeking | 333 | 8,919 | 0.04 | 0.03–0.04 |
| Offline medication purchase | AI-assisted consultation | 105 | 8,919 | 0.01 | 0.01–0.01 |
| Offline medication purchase | Telemedicine | 73 | 8,919 | 0.01 | 0.01–0.01 |
| Offline medication purchase | Online medication purchase | 0 | 8,919 | 0.00 | 0.00–0.00 |
| Offline medication purchase | Wearable device–based self-monitoring | 34 | 8,919 | 0.00 | 0.00–0.01 |
| Offline medication purchase | Self-administered rapid antigen testing | 58 | 8,919 | 0.01 | 0.00–0.01 |
| Offline medication purchase | At-home pathogen specimen collection | 5 | 8,919 | 0.00 | 0.00–0.00 |
| Offline medication purchase | No subsequent reported action | 6,967 | 8,919 | 0.78 | 0.77–0.79 |
| Online medication purchase | In-person facility attendance | 405 | 2,736 | 0.15 | 0.14–0.16 |
| Online medication purchase | Online health information seeking | 145 | 2,736 | 0.05 | 0.04–0.06 |
| Online medication purchase | AI-assisted consultation | 44 | 2,736 | 0.02 | 0.01–0.02 |
| Online medication purchase | Telemedicine | 34 | 2,736 | 0.01 | 0.01–0.02 |
| Online medication purchase | Offline medication purchase | 0 | 2,736 | 0.00 | 0.00–0.00 |
| Online medication purchase | Wearable device–based self-monitoring | 16 | 2,736 | 0.01 | 0.00–0.01 |
| Online medication purchase | Self-administered rapid antigen testing | 54 | 2,736 | 0.02 | 0.01–0.03 |
| Online medication purchase | At-home pathogen specimen collection | 8 | 2,736 | 0.00 | 0.00–0.01 |
| Online medication purchase | No subsequent reported action | 2,030 | 2,736 | 0.74 | 0.72–0.76 |
| Wearable device–based self-monitoring | In-person facility attendance | 171 | 1,205 | 0.14 | 0.12–0.16 |
| Wearable device–based self-monitoring | Online health information seeking | 63 | 1,205 | 0.05 | 0.04–0.06 |
| Wearable device–based self-monitoring | AI-assisted consultation | 43 | 1,205 | 0.04 | 0.03–0.05 |
| Wearable device–based self-monitoring | Telemedicine | 40 | 1,205 | 0.03 | 0.02–0.04 |
| Wearable device–based self-monitoring | Offline medication purchase | 122 | 1,205 | 0.10 | 0.09–0.12 |
| Wearable device–based self-monitoring | Online medication purchase | 56 | 1,205 | 0.05 | 0.04–0.06 |
| Wearable device–based self-monitoring | Self-administered rapid antigen testing | 17 | 1,205 | 0.01 | 0.01–0.02 |
| Wearable device–based self-monitoring | At-home pathogen specimen collection | 11 | 1,205 | 0.01 | 0.00–0.01 |
| Wearable device–based self-monitoring | No subsequent reported action | 682 | 1,205 | 0.57 | 0.54–0.59 |
| Self-administered rapid antigen testing | In-person facility attendance | 173 | 932 | 0.19 | 0.16–0.21 |
| Self-administered rapid antigen testing | Online health information seeking | 34 | 932 | 0.04 | 0.03–0.05 |
| Self-administered rapid antigen testing | AI-assisted consultation | 19 | 932 | 0.02 | 0.01–0.03 |
| Self-administered rapid antigen testing | Telemedicine | 20 | 932 | 0.02 | 0.01–0.03 |
| Self-administered rapid antigen testing | Offline medication purchase | 38 | 932 | 0.04 | 0.03–0.05 |
| Self-administered rapid antigen testing | Online medication purchase | 35 | 932 | 0.04 | 0.03–0.05 |
| Self-administered rapid antigen testing | Wearable device–based self-monitoring | 9 | 932 | 0.01 | 0.00–0.02 |
| Self-administered rapid antigen testing | At-home pathogen specimen collection | 6 | 932 | 0.01 | 0.00–0.01 |
| Self-administered rapid antigen testing | No subsequent reported action | 598 | 932 | 0.64 | 0.61–0.67 |
| At-home pathogen specimen collection | In-person facility attendance | 28 | 218 | 0.13 | 0.09–0.18 |
| At-home pathogen specimen collection | Online health information seeking | 14 | 218 | 0.06 | 0.03–0.10 |
| At-home pathogen specimen collection | AI-assisted consultation | 7 | 218 | 0.03 | 0.01–0.06 |
| At-home pathogen specimen collection | Telemedicine | 16 | 218 | 0.07 | 0.04–0.11 |
| At-home pathogen specimen collection | Offline medication purchase | 7 | 218 | 0.03 | 0.01–0.06 |
| At-home pathogen specimen collection | Online medication purchase | 5 | 218 | 0.02 | 0.00–0.04 |
| At-home pathogen specimen collection | Wearable device–based self-monitoring | 5 | 218 | 0.02 | 0.00–0.05 |
| At-home pathogen specimen collection | Self-administered rapid antigen testing | 13 | 218 | 0.06 | 0.03–0.09 |
| At-home pathogen specimen collection | No subsequent reported action | 123 | 218 | 0.56 | 0.50–0.63 |

**Note.** Probabilities are empirical adjacent-transition probabilities. Self-transitions were structurally precluded because each behavior could be dated only once per episode. Each origin-state row sums to approximately 1.00 after including the terminal state. CIs were obtained from 2000 participant-level bootstrap iterations, retaining all transitions contributed by each resampled participant. “No subsequent reported action” indicates only that no later dated action was recorded; it does not indicate recovery or successful self-management.

## **Table S7. Sequence inclusion, participant-level transition contributions, and most frequently reported complete behavioral sequences.**

**Part A. Sequence inclusion and transition contributions**

| **Sequence-analysis item** | **n** | **% of all participants with recent ARI symptoms** |
| --- | --- | --- |
| All participants with recent ARI symptoms | 68,650 | 100.00 |
| Participants with at least 1 dated representative action | 26,494 | 38.59 |
| Participants with at least 2 dated representative actions | 6,631 | 9.66 |
| Participants contributing more than 1 transition when terminal is included | 6,631 | 9.66 |
| Participants with multiple actions reported on the same day | 9,987 | 14.55 |
| Maximum transitions contributed by 1 participant | 6 |  |

**Distribution of transitions per participant**

| **Transitions contributed, n** | **Participants, n** | **% of nonempty sequences** |
| --- | --- | --- |
| 1 | 19,863 | 74.97 |
| 2 | 5,635 | 21.27 |
| 3 | 850 | 3.21 |
| 4 | 131 | 0.49 |
| 5 | 12 | 0.05 |
| 6 | 3 | 0.01 |

**Part B. Most frequently reported complete sequences**

| **Rank** | **Complete sequence** | **n** | **% of nonempty sequences** |
| --- | --- | --- | --- |
| 1 | In-person facility attendance → No subsequent reported action | 8,352 | 31.52 |
| 2 | Offline medication purchase → No subsequent reported action | 6,058 | 22.87 |
| 3 | Online medication purchase → No subsequent reported action | 1,601 | 6.04 |
| 4 | Online health information seeking → No subsequent reported action | 1,369 | 5.17 |
| 5 | Offline medication purchase → In-person facility attendance → No subsequent reported action | 1,202 | 4.54 |
| 6 | AI-assisted consultation → No subsequent reported action | 873 | 3.30 |
| 7 | Telemedicine → No subsequent reported action | 686 | 2.59 |
| 8 | Wearable device–based self-monitoring → No subsequent reported action | 508 | 1.92 |
| 9 | In-person facility attendance → Offline medication purchase → No subsequent reported action | 389 | 1.47 |
| 10 | Online health information seeking → In-person facility attendance → No subsequent reported action | 354 | 1.34 |
| 11 | Self-administered rapid antigen testing → No subsequent reported action | 347 | 1.31 |
| 12 | Online medication purchase → In-person facility attendance → No subsequent reported action | 324 | 1.22 |
| 13 | In-person facility attendance → Online health information seeking → No subsequent reported action | 282 | 1.06 |
| 14 | Offline medication purchase → Online health information seeking → No subsequent reported action | 245 | 0.92 |
| 15 | Online health information seeking → Offline medication purchase → No subsequent reported action | 208 | 0.79 |

**Note.** All percentages in the transition distribution and complete-sequence ranking use the 26,494 participants with a nonempty sequence as the denominator, unless explicitly labeled as a percentage of all 68,650 participants with recent ARI symptoms. A terminal “No subsequent reported action” state was appended to every nonempty sequence.

**Table S8. Sensitivity analyses for digital health classification and model specification.**

**Part A. Digital health classification**

| **Definition** | **Outcome** | **n/N** | **Weighted % (95% CI)** |
| --- | --- | --- | --- |
| Primary channel-based definition | Any digital health engagement | 13,684/68,650 | 18.94% (95% CI 18.64–19.24) |
| Primary channel-based definition | In-person facility attendance with digital health engagement | 5,545/68,650 | 7.74% (95% CI 7.54–7.95) |
| Primary channel-based definition | Digital health engagement without in-person facility attendance | 8,139/68,650 | 11.20% (95% CI 10.95–11.44) |
| Primary channel-based definition | In-person facility attendance without digital health engagement | 7,370/68,650 | 11.43% (95% CI 11.19–11.68) |
| Primary channel-based definition | Neither measured dimension | 47,596/68,650 | 69.63% (95% CI 69.27–69.98) |
| Broad definition: all self-administered rapid antigen testing classified as digital | Any digital health engagement | 13,976/68,650 | 19.37% (95% CI 19.07–19.68) |
| Broad definition: all self-administered rapid antigen testing classified as digital | In-person facility attendance with digital health engagement | 5,716/68,650 | 8.00% (95% CI 7.79–8.21) |
| Broad definition: all self-administered rapid antigen testing classified as digital | Digital health engagement without in-person facility attendance | 8,260/68,650 | 11.37% (95% CI 11.13–11.62) |
| Broad definition: all self-administered rapid antigen testing classified as digital | In-person facility attendance without digital health engagement | 7,199/68,650 | 11.18% (95% CI 10.94–11.42) |
| Broad definition: all self-administered rapid antigen testing classified as digital | Neither measured dimension | 47,475/68,650 | 69.45% (95% CI 69.09–69.80) |
| Ambiguous self-testing channels classified as nondigital | Any digital health engagement | 13,684/68,650 | 18.94% (95% CI 18.64–19.24) |
| Ambiguous self-testing channels classified as nondigital | In-person facility attendance with digital health engagement | 5,545/68,650 | 7.74% (95% CI 7.54–7.95) |
| Ambiguous self-testing channels classified as nondigital | Digital health engagement without in-person facility attendance | 8,139/68,650 | 11.20% (95% CI 10.95–11.44) |
| Ambiguous self-testing channels classified as nondigital | In-person facility attendance without digital health engagement | 7,370/68,650 | 11.43% (95% CI 11.19–11.68) |
| Ambiguous self-testing channels classified as nondigital | Neither measured dimension | 47,596/68,650 | 69.63% (95% CI 69.27–69.98) |
| Ambiguous self-testing channels classified as digital | Any digital health engagement | 13,700/68,650 | 18.97% (95% CI 18.67–19.27) |
| Ambiguous self-testing channels classified as digital | In-person facility attendance with digital health engagement | 5,556/68,650 | 7.76% (95% CI 7.56–7.97) |
| Ambiguous self-testing channels classified as digital | Digital health engagement without in-person facility attendance | 8,144/68,650 | 11.21% (95% CI 10.96–11.45) |
| Ambiguous self-testing channels classified as digital | In-person facility attendance without digital health engagement | 7,359/68,650 | 11.42% (95% CI 11.17–11.66) |
| Ambiguous self-testing channels classified as digital | Neither measured dimension | 47,591/68,650 | 69.62% (95% CI 69.26–69.97) |

**Self-testing acquisition channels**

| **Acquisition channel** | **n** | **Denominator** | **%** |
| --- | --- | --- | --- |
| Self-testing total | 2,203 | 2,203 | 100.00 |
| Offline pharmacy only | 1,121 | 2,203 | 50.89 |
| Online only | 783 | 2,203 | 35.54 |
| Both online and offline | 260 | 2,203 | 11.80 |
| Any online channel | 1,043 | 2,203 | 47.34 |
| Channel missing or ambiguous | 39 | 2,203 | 1.77 |

**Part B. Regression specification**

| **Model** | **Measure** | **Estimate (95% CI)** | **Analysis n** | **Variance estimation** |
| --- | --- | --- | --- | --- |
| Primary modified Poisson model | aPR | 2.12 (95% CI 2.05–2.20) | 68,650 | Individual-level robust |
| Broad-definition modified Poisson model | aPR | 2.17 (95% CI 2.10–2.25) | 68,650 | Individual-level robust |
| Weighted logistic regression sensitivity analysis | aOR | 3.18 (95% CI 3.02–3.35) | 68,650 | Individual-level robust |

**Adjusted marginal estimates from the primary model**

| **Estimand** | **Estimate (95% CI)** |
| --- | --- |
| Adjusted prevalence of in-person facility attendance: exposure=0 | 15.08% (95% CI 14.78–15.39) |
| Adjusted prevalence of in-person facility attendance: exposure=1 | 32.01% (95% CI 31.17–32.84) |
| Adjusted prevalence difference: exposure=1 minus exposure=0 | 16.92 percentage points (95% CI 16.02–17.83) |

**Note.** For the descriptive weighted proportions in Part A, 95% CIs are Wilson score intervals based on the Kish effective sample size derived from poststratification weights. The regression models in Part B used poststratification weights and individual-level robust (sandwich) variance estimation. The weighted logistic model reports an aOR, not a PR. Adjusted marginal estimates average model predictions over the observed weighted covariate distribution, with delta-method CIs based on the individual-level robust covariance matrix. aOR indicates adjusted odds ratio; aPR, adjusted prevalence ratio; CI, confidence interval.

## **Table S9. Survey-weighted prevalence of digital health engagement, in-person facility attendance, and specific behaviors using an alternative adult age categorization.**

| **Outcome** | **<18 years, n** | **<18 years, weighted % (95% CI)** | **18–39 years, n** | **18–39 years, weighted % (95% CI)** | **40–59 years, n** | **40–59 years, weighted % (95% CI)** | **≥60 years, n** | **≥60 years, weighted % (95% CI)** |
| --- | --- | --- | --- | --- | --- | --- | --- | --- |
| Any digital health engagement | 2,249 | 14.45% (95% CI 13.90–15.02) | 6,149 | 25.76% (95% CI 25.20–26.32) | 4,461 | 20.50% (95% CI 19.96–21.04) | 825 | 10.44% (95% CI 9.78–11.14) |
| In-person facility attendance | 3,253 | 21.27% (95% CI 20.63–21.92) | 3,961 | 16.70% (95% CI 16.23–17.18) | 4,011 | 18.59% (95% CI 18.08–19.12) | 1,690 | 21.80% (95% CI 20.90–22.73) |
| In-person facility attendance with digital health engagement | 1,077 | 6.97% (95% CI 6.58–7.38) | 2,299 | 9.62% (95% CI 9.25–10.01) | 1,792 | 8.24% (95% CI 7.88–8.62) | 377 | 4.79% (95% CI 4.34–5.29) |
| Digital health engagement without in-person facility attendance | 1,172 | 7.48% (95% CI 7.08–7.91) | 3,850 | 16.13% (95% CI 15.67–16.61) | 2,669 | 12.25% (95% CI 11.82–12.70) | 448 | 5.65% (95% CI 5.16–6.19) |
| In-person facility attendance without digital health engagement | 2,176 | 14.30% (95% CI 13.75–14.86) | 1,662 | 7.07% (95% CI 6.75–7.41) | 2,219 | 10.35% (95% CI 9.95–10.76) | 1,313 | 17.01% (95% CI 16.19–17.86) |
| Neither measured dimension | 11,128 | 71.25% (95% CI 70.53–71.96) | 15,854 | 67.17% (95% CI 66.57–67.77) | 14,909 | 69.16% (95% CI 68.53–69.77) | 5,705 | 72.55% (95% CI 71.54–73.53) |
| Online health information seeking | 1,237 | 7.96% (95% CI 7.55–8.40) | 4,351 | 18.24% (95% CI 17.75–18.74) | 2,988 | 13.75% (95% CI 13.29–14.22) | 463 | 5.83% (95% CI 5.33–6.37) |
| AI-assisted consultation | 505 | 3.24% (95% CI 2.97–3.53) | 1,600 | 6.74% (95% CI 6.42–7.06) | 1,252 | 5.79% (95% CI 5.49–6.11) | 212 | 2.71% (95% CI 2.37–3.09) |
| Telemedicine | 526 | 3.39% (95% CI 3.11–3.68) | 1,179 | 4.97% (95% CI 4.70–5.25) | 938 | 4.33% (95% CI 4.07–4.62) | 254 | 3.27% (95% CI 2.89–3.69) |
| Offline medication purchase | 2,536 | 16.37% (95% CI 15.79–16.97) | 4,467 | 18.93% (95% CI 18.43–19.43) | 4,875 | 22.62% (95% CI 22.07–23.19) | 1,552 | 19.97% (95% CI 19.10–20.88) |
| Online medication purchase | 885 | 5.70% (95% CI 5.34–6.08) | 2,167 | 8.96% (95% CI 8.60–9.33) | 1,209 | 5.44% (95% CI 5.14–5.75) | 181 | 2.23% (95% CI 1.93–2.58) |
| Wearable device–based self-monitoring | 366 | 2.32% (95% CI 2.09–2.57) | 1,301 | 5.47% (95% CI 5.19–5.77) | 815 | 3.78% (95% CI 3.53–4.04) | 153 | 1.96% (95% CI 1.67–2.29) |
| Self-administered rapid antigen testing | 452 | 2.91% (95% CI 2.65–3.19) | 986 | 4.12% (95% CI 3.88–4.39) | 623 | 2.86% (95% CI 2.65–3.10) | 142 | 1.82% (95% CI 1.54–2.14) |
| At-home pathogen specimen collection | 177 | 1.15% (95% CI 0.99–1.33) | 413 | 1.74% (95% CI 1.58–1.92) | 261 | 1.21% (95% CI 1.08–1.37) | 64 | 0.83% (95% CI 0.65–1.05) |

**Note.** Digital composite outcomes use the primary channel-based definition. The row labeled self-administered rapid antigen testing reports all such testing; only tests obtained through an e-commerce or on-demand delivery platform contributed to the digital composite. At-home pathogen specimen collection was classified as digitally arranged. Values are unweighted numbers and survey-weighted proportions. Weighted 95% CIs are Wilson score intervals based on the Kish effective sample size derived from poststratification weights within each age subgroup.

## **Table S10. Modified Poisson regression using the alternative adult age categorization.**

| **Variable** | **Reference category** | **aPR** | **95% CI** |
| --- | --- | --- | --- |
| Digital health engagement: yes vs no | No digital health engagement | 2.15 | 2.08–2.22 |
| Age 18–39 years vs <18 years | <18 years | 0.69 | 0.65–0.72 |
| Age 40–59 years vs <18 years | <18 years | 0.80 | 0.76–0.84 |
| Age ≥60 years vs <18 years | <18 years | 0.97 | 0.92–1.03 |
| Female vs male | Male | 1.06 | 1.03–1.09 |
| Rural vs urban residence | Urban | 1.16 | 1.13–1.20 |
| Middle vs high regional GDP level | High GDP | 0.71 | 0.68–0.73 |
| Low vs high regional GDP level | High GDP | 1.21 | 1.16–1.26 |
| Smartphone owner vs non-owner | Non-owner | 0.93 | 0.89–0.98 |
| 3 symptoms vs ≤2 symptoms | ≤2 symptoms | 2.83 | 2.70–2.97 |
| ≥4 symptoms vs ≤2 symptoms | ≤2 symptoms | 3.71 | 3.55–3.87 |

**Note.** The outcome was in-person facility attendance. The overall digital model used modified Poisson regression with a log link, poststratification weights, and individual-level robust (sandwich) variance estimation. The model included digital health engagement under the primary channel-based definition, alternative age group, sex, urban-rural residence, regional GDP level, smartphone ownership, and symptom-count category. Estimates are cross-sectional associations. aPR indicates adjusted prevalence ratio; CI, confidence interval; GDP, gross domestic product.
